# ART in Europe, 2018: results generated from European registries by ESHRE^†^

**DOI:** 10.1093/hropen/hoac022

**Published:** 2022-07-05

**Authors:** European IVF Monitoring Consortium (EIM), for the European Society of Human Reproduction and Embryology (ESHRE), Orion Gliozheni, Eduard Hambartsoumian, Heinz Strohmer, Elena Petrovskaya, Oleg Tishkevich, Diane de Neubourg, Kris Bogaerts, Devleta Balic, Sanja Sibincic, Irena Antonova, Hrvoje Vrcic, Dejan Ljiljak, Karel Rezabek, Jitka Markova, John Kirk, Deniss Sõritsa, Mika Gissler, Sari Pelkonen, Jacques de Mouzon, Andreas Tandler, Nikos Vrachnis, Janos Urbancsek, G Kosztolanyi, Hilmar Bjorgvinsson, Mary Wingfield, Joyce Leyden, Giulia Scaravelli, Roberto de Luca, Vyacheslav Lokshin, Sholpan Karibayeva, Valeria Magomedova, Raminta Bausyte, Ieva Masliukaite, Caroline Schilling, Jean Calleja-Agius, Veaceslav Moshin, Tatjana Motrenko Simic, Dragana Vukicevic, Jesper M J Smeenk, Zoranco Petanovski, Liv Bente Romundstad, Anna Janicka, Carlos Calhaz-Jorge, Joana Maria Mesquita Guimaraes, Ana Rita Laranjeira, Ioana Rugescu, Bogdan Doroftei, Vladislav Korsak, Snezana Vidakovic, Borut Kovacic, Irene Cuevas Sáiz, Fernando Prados Mondéjar, Christina Bergh, Maya Weder, Marco Buttarelli, Mete Isikoglu, Basak Balaban, Richard Baranowski, Mykola Gryshchenko, C Wyns, C De Geyter, C Calhaz-Jorge, M S Kupka, T Motrenko, J Smeenk, C Bergh, A Tandler-Schneider, I A Rugescu, V Goossens

**Affiliations:** Cliniques Universitaires Saint-Luc, Université Catholique de Louvain, Brussels, Belgium; Reproductive Medicine and Gynecological Endocrinology (RME), University Hospital, University of Basel, Basel, Switzerland; Faculdade de Medicina da Universidade de Lisboa, Lisbon, Portugal; Fertility Center—Gynaekologicum, Hamburg, Germany; Human Reproduction Center Budva, Budva, Montenegro; Elisabeth Twee Steden Ziekenhuis, Tilburg, The Netherlands; Department of Obstetrics and Gynecology, Institute of Clinical Sciences, Göteborg University, Göteborg, Sweden; Fertility Center Berlin, Berlin, Germany; National Transplant Agency, Bucharest, Romania; ESHRE Central Office, Strombeek-Bever, Belgium

**Keywords:** IVF, ICSI, IUI, egg donation, frozen embryo transfer, surveillance, vigilance, registry, data collection, fertility preservation

## Abstract

**STUDY QUESTION:**

What are the data and trends on ART and IUI cycle numbers and their outcomes, and on fertility preservation (FP) interventions, reported in 2018 as compared to previous years?

**SUMMARY ANSWER:**

The 22nd ESHRE report shows a continued increase in reported numbers of ART treatment cycles and children born in Europe, a decrease in transfers with more than one embryo with a further reduction of twin delivery rates (DRs) as compared to 2017, higher DRs per transfer after fresh IVF or ICSI cycles (without considering freeze-all cycles) than after frozen embryo transfer (FET) with higher pregnancy rates (PRs) after FET and the number of reported IUI cycles decreased while their PR and DR remained stable.

**WHAT IS KNOWN ALREADY:**

ART aggregated data generated by national registries, clinics or professional societies have been gathered and analysed by the European IVF-monitoring Consortium (EIM) since 1997 and reported in 21 manuscripts published in *Human Reproduction* and *Human Reproduction Open.*

**STUDY DESIGN, SIZE, DURATION:**

Data on medically assisted reproduction (MAR) from European countries are collected by EIM for ESHRE on a yearly basis. The data on treatment cycles performed between 1 January and 31 December 2018 were provided by either national registries or registries based on initiatives of medical associations and scientific organizations or committed persons of 39 countries.

**PARTICIPANTS/MATERIALS, SETTING, METHODS:**

Overall, 1422 clinics offering ART services in 39 countries reported a total of more than 1 million (1 007 598) treatment cycles for the first time, including 162 837 with IVF, 400 375 with ICSI, 309 475 with FET, 48 294 with preimplantation genetic testing, 80 641 with egg donation (ED), 532 with IVM of oocytes and 5444 cycles with frozen oocyte replacement (FOR). A total of 1271 institutions reported data on IUI cycles using either husband/partner’s semen (IUI-H; n = 148 143) or donor semen (IUI-D; n = 50 609) in 31 countries and 25 countries, respectively. Sixteen countries reported 20 994 interventions in pre- and post-pubertal patients for FP including oocyte, ovarian tissue, semen and testicular tissue banking.

**MAIN RESULTS AND THE ROLE OF CHANCE:**

In 21 countries (21 in 2017) in which all ART clinics reported to the registry, 410 190 treatment cycles were registered for a total population of ∼ 300 million inhabitants, allowing a best estimate of a mean of 1433 cycles performed per million inhabitants (range: 641–3549). Among the 39 reporting countries, for IVF, the clinical PR per aspiration slightly decreased while the PR per transfer remained similar compared to 2017 (25.5% and 34.1% in 2018 versus 26.8% and 34.3% in 2017). In ICSI, the corresponding rates showed similar evolutions in 2018 compared to 2017 (22.5% and 32.1% in 2018 versus 24.0% and 33.5% in 2017). When freeze-all cycles were not considered for the calculations, the clinical PRs per aspiration were 28.8% (29.4% in 2017) and 27.3% (27.3% in 2017) for IVF and ICSI, respectively. After FET with embryos originating from own eggs, the PR per thawing was 33.4% (versus 30.2% in 2017), and with embryos originating from donated eggs 41.8% (41.1% in 2017). After ED, the PR per fresh embryo transfer was 49.6% (49.2% in 2017) and per FOR 44.9% (43.3% in 2017). In IVF and ICSI together, the trend towards the transfer of fewer embryos continues with the transfer of 1, 2, 3 and ≥4 embryos in 50.7%, 45.1%, 3.9% and 0.3% of all treatments, respectively (corresponding to 46.0%, 49.2%. 4.5% and 0.3% in 2017). This resulted in a reduced proportion of twin DRs of 12.4% (14.2% in 2017) and similar triplet DR of 0.2%. Treatments with FET in 2018 resulted in twin and triplet DRs of 9.4% and 0.1%, respectively (versus 11.2% and 0.2%, respectively in 2017). After IUI, the DRs remained similar at 8.8% after IUI-H (8.7% in 2017) and at 12.6% after IUI-D (12.4% in 2017). Twin and triplet DRs after IUI-H were 8.4% and 0.3%, respectively (in 2017: 8.1% and 0.3%), and 6.4% and 0.2% after IUI-D (in 2017: 6.9% and 0.2%). Among 20 994 FP interventions in 16 countries (18 888 in 13 countries in 2017), cryopreservation of ejaculated sperm (n = 10 503, versus 11 112 in 2017) and of oocytes (n = 9123 versus 6588 in 2017) were the most frequently reported.

**LIMITATIONS, REASONS FOR CAUTION:**

The results should be interpreted with caution as data collection systems and completeness of reporting vary among European countries. Some countries were unable to deliver data about the number of initiated cycles and/or deliveries.

**WIDER IMPLICATIONS OF THE FINDINGS:**

The 22nd ESHRE data collection on ART, IUI and FP interventions shows a continuous increase of reported treatment numbers and MAR-derived livebirths in Europe. Although it is the largest data collection on MAR in Europe, further efforts towards optimization of both the collection and reporting, with the aim of improving surveillance and vigilance in the field of reproductive medicine, are awaited.

**STUDY FUNDING/COMPETING INTEREST(S):**

The study has received no external funding and all costs are covered by ESHRE. There are no competing interests.

## Introduction

This is the 22nd annual report of the European IVF-monitoring Consortium (EIM) under the umbrella of ESHRE, assembling the data on ART, IUI and fertility preservation (FP) as reported by 39 participating European countries in 2018 (Supplementary Table SI and Supplementary Data).

Eighteen previous annual reports published in *Human Reproduction* (https://www.eshre.eu/Data-collection-and-research/Consortia/EIM/Publications.aspx) and three in *Human Reproduction Open* (De Geyter *et al.*, 2020a; Wyns *et al.*, 2020, 2021) covered data on treatment cycles collected on a yearly basis from 1997 to 2017. As in previous reports, the printed version contains the five most relevant tables. Twenty additional supplementary tables (Supplementary Tables SI–SXX) are available online on the publisher’s homepage. To allow easy comparison and assessment of trends, the presentation of the data is consistent with previous reports. For the third consecutive year, data on FP were collected and added to this report.

## Materials and methods

Data were collected on an aggregate basis and were provided by 39 European countries, covering treatments with IVF, ICSI, frozen embryo transfer (FET), egg donation (ED), IVM, preimplantation genetic testing (PGT; pooled data), frozen oocyte replacement (FOR), IUI with husband’s/partner’s semen (IUI-H) and with donor semen (IUI-D). The report includes treatments started between 1 January and 31 December in 2018. Data on pregnancies and deliveries represent the outcomes of treatments performed in 2018. Aggregated data on FP include numbers and types of cryopreserved material and interventions for use of cryostored material between 1 January and 31 December in 2018.

The national representatives of the 44 countries that are members of the EIM consortium were asked to fill out the survey with the same data requirements as in 2017. A total of 10 modules on specific topics/questions were sent using software designed for the requirements of this data collection (Dynamic Solutions, Barcelona, Spain). Any identified inconsistency was clarified through direct contacts between the administrator of the ESHRE central office (V.G.) and the national representative.

The data were analysed and presented similarly to previous reports. Footnotes to the tables were added to clarify some results reported by individual countries, when applicable.

The terminology used was based on the glossary of The International Committee for Monitoring Assisted Reproductive Technology (Zegers-Hochschild *et al.*, 2017).

## Results

### Participation and data completeness

Table I shows the number of clinics providing ART services with the different treatment modalities they offer and institutions performing IUI (IUI-H and IUI-D). Compared to 2017, the total number of reporting clinics (1422 versus 1381 in 2017) and number of reported treatments (1 007 598 versus 940 503 in 2017, +7.1%) increased. Among the 51 European countries, 44 are EIM members including 28 that were members of the European Union (EU) at that time and 39 (39 in 2017) provided data (Supplementary Table SI). Non-EIM members are mainly small countries not offering ART services. Croatia, Cyprus, Georgia, Slovakia and Turkey did not deliver data in 2018 (11.4% of EIM members, as in 2017, but with Croatia failing to provide data and Ireland sending data). In 21 countries (53.8% of reporting countries as in 2017), all ART clinics participated in the reporting. Among 1552 known IVF clinics in Europe, 1422 clinics reported data sets (91.6% versus 90.1% in 2017). As in 2017, the four European countries with the largest treatment numbers in 2018 were Russia (155 949; 137 211 in 2017), Spain (140 498; 125 592 in 2017), France (106 884; 108 820 in 2017) and Germany (105 328; 99 466 in 2017).

**Table I:** Treatment frequencies after ART in European countries in 2018.

	IVF clinics in the country												Cycles/million*	
Country	IVF clinics	Included IVF clinics	IUI labs	Included IUI labs	IVF	ICSI	FET	PGT	ED	IVM	FOR	All	Women 15–45	Population
Albania	8	1	10	1	0	86	69	21	24	0	2	202		
Armenia	7	6	10	6	786	967	2156	24	815			4748		
Austria	30	30	0	0	1701	5703	3161	0	61			10 626	6611	1209
Belarus	8	7	10	7	1253	2039	992	69	115		1	4469		
Belgium	18	18	28	28	2554	13 783	13 821	1397	1603	167	91	33 416	15 449	2891
Bosnia-Herzegovina, Federation part	10	1	10	1	0	111	58	0	0			169		
Bulgaria	37	37	38	38	1078	8404	2469	0	1140			13 091	10 580	1855
Czech Republic	48	48	0	0	1954	13 481	14 749	0	5007			35 191	18 310	3294
Denmark	19	19	54	52	6479	5536	6912	536	1120	0	33	20 616	19 181	3549
Estonia	6	6	6	6	672	1394	1044	0	212	0	4	3326	15 948	2674
Finland	16	16	20	20	2538	1834	3736	279	714			9101	9241	1643
France	103	102	180	179	20 821	41 021	41 258	1646	1532	127	479	106 884		
Germany	140	134	0	0	21 007	54 719	29 334		0		268	105 328		
Greece	43	33	0	31	2111	14 174	5623	575	4875	20	76	27 454		
Hungary	14	11	21	13		7819	1186		0			9005		
Iceland	1	1	1	1	301	188	307	0	101	0	1	898	12 910	2614
Ireland	9	2	2	1	291	316	408	609	2	0	3	1629		
Italy	202	202	345	345	7871	43 215	19 587	3441	5947		1318	81 379	7650	1309
Kazakhstan	8	8	0	0	1555	4955	3489	829	1187			12 015	2878	641
Latvia	6	3	6	3	143	439	359	46	83	0	1	1071		
Lithuania	6	6	6	6	472	405	201	7	3	0	0	1088	2335	389
Luxembourg	1	1	0	0	175	560	397		0	0	0	1132	8760	1868
Malta	2	2	2	0	0	249	2	0	0	0	73	324	3769	722
Moldova	4	3	5	3	0	1248	210		0			1458		
Montenegro	5	4	5	4	0	586	57		0			643		
North-Macedonia	7	5	1	0	438	2199	411	0	151	0	0	3199		
Norway	11	11	11	11	4208	3161	4178	0	0			11 547	11 109	2150
Poland	45	45	0	42	263	16 221	12 423	1545	1489	7	375	32 323	4365	841
Portugal	25	25	27	27	2476	3740	2725	436	1496	9	80	10 962	5890	1060
Romania	20	12	20	12	1696	2303	2174	20	0		20	6213		
Russia	230	182	0	0	38 937	53 609	43 996	8783	9804	87	733	155 949		
Serbia	18	1	18	1	102	48	16	0	0	0	2	168		
Slovenia	3	3	2	2	1213	2181	1568	85	4	0	10	5061	14622	2408
Spain	247	245	366	313	6352	42 773	31 894	20 783	37 618	9	1069	140 498		
Sweden	18	18	0	0	5990	5733	7167	509	346			19 745	10 621	1968
Switzerland	28	28	0	0	920	5450	4871	356	0			11 597	7351	1399
The Netherlands	15	15	0	0	6363	7082	13 342	414	0			27 201	8721	1587
Ukraine	48	45	17	17	628	11 410	10 620	4098	1552		13	28 321		
UK	86	86	101	101	19 489	21 233	22 505	1786	3640	106	792	69 551	5728	1069
**All**	1552	1422	1322	1271	162837	400375	309475	48294	80641	532	5444	1007598	7581	1402

Treatment cycles in IVF and ICSI refer to initiated cycles.

For Austria, Belgium, Denmark and France, treatment cycles refer to aspirations. For Austria and Belgium, the total number of initiated cycles was only available for IVF and ICSI together, being 10 828 and 19 032. For Hungary and Malta, the number refers to aspiration cycles for IVF + ICSI.

For Belgium, there were 875 extra aspiration cycles for which it is not known whether IVF or ICSI was performed. From these, only one had a transfer without pregnancy.

Treatment cycles in frozen embryo transfer (FET) refer to thawings.

For Finland, Hungary, Kazakhstan, Luxembourg, Moldova, Sweden and the Netherlands, treatment cycles refer to transfers.

Treatment cycles in PGT contain both fresh and frozen cycles and refer to initiated cycles in the fresh cycles and thawings in the frozen cycles.

For Finland, it refers to initiated cycles and transfers; for France, it refers to aspirations and thawings. Kazakhstan and Lithuania only reported fresh, initiated cycles.

Treatment cycles in egg donation (ED) refer to donation cycles and contain fresh and frozen cycles.

Treatment cycles in IVM refer to aspirations.

Treatment cycles in frozen oocyte replacement (FOR) refer to thawings.

PGT, preimplantation genetic testing.

### Size of the clinics and reporting methods

The size of reporting clinics, as calculated based on the number of cycles per year, was highly variable among and within countries, as seen in previous years (Supplementary Table SII). In 2018, clinics with cycle numbers between 200 and 499 and 500–999 were the most common (27.3% and 26.3%). These numbers were comparable with the numbers reported in 2017 (25.9% and 26.3%). The proportion of clinics performing more than 1000 treatment cycles per year was slightly higher than in 2017 (21% versus 19% in 2017). Small clinics with <100 treatment cycles per year were present in 21 countries (24 countries in 2017).

Country-specific requirements of registries and reporting methods are shown in Supplementary Table SIII. Data collection was either voluntary (16 out of 39 countries) or compulsory. Twenty-two countries reported all or a part of the treatment cycles to the national health authority. Among 18 countries with only partial reporting, the data were mainly provided on a voluntary basis (12 countries) to medical organizations (7 countries), to the national health authority (8 countries) or as a single person who took the initiative (3 countries).

In contrast, complete reporting was most often achieved when data collection was compulsory (17/21 countries) with data communication to the national health authority (all but 4 countries). Transfer of the data was mainly done on an aggregate basis (25 countries/39).

### Number of treatment cycles per technique and availability

In 2018, 1 007 598 treatment cycles were reported to EIM (67 095 more than in 2017, +7.1%). Since 1997, increasing numbers of clinics reported to EIM to reach a total 11 726 598 treatment cycles and the birth of more than 2 275 585 infants (Table II). As seen in Table I, 11 countries reported fewer treatment cycles than in 2017. Furthermore, the largest increments in reported treatment numbers were observed for Russia (+ 18 738, +23 clinics) and Spain (+14 906, +6 clinics). Table I shows the numbers of treatment cycles per technique in 2018: ICSI remains the most used (400 375, 39.7% of all treatment cycles versus 391 379, 41.6% in 2017). Cycles of IVF, FET, ED, FOR, PGT and IVM represented 16.2%, 30.7%, 8%, 0.5%, 4.8% and 0.0005% of all cycles, respectively. The distribution of the available techniques remained similar to 2017 (respectively, 17.6%, 28.9%, 7.4%, 0.5%, 4% and 0.0004% for IVF, FET, ED, FOR, PGT and IVM); however, reported cycle numbers increased for ICSI, FET, ED, PGT, IVM and FOR, and decreased for IVF (–1.5%).

**Table II:** Number of institutions offering ART services, treatment cycles and infants born after ART in Europe, 1997–2018.

Year	No. of countries	No. of centres	No. of cycles	Cycle increase (%)	No. of infants born
1997	18	482	203 225		35 314
1998	18	521	232 225	+14.3	21 433
1999	21	537	249 624	+7.5	26 212
2000	22	569	275 187	+10.2	17 887
2001	23	579	289 690	+5.3	24 963
2002	25	631	324 238	+11.9	24 283
2003	28	725	365 103	+12.6	68 931
2004	29	785	367 056	+0.5	67 973
2005	30	923	419 037	+14.2	72 184
2006	32	998	458 759	+9.5	87 705
2007	33	1029	493 420	+7.7	96 690
2008	36	1051	532 260	+7.9	107 383
2009	34	1005	537 463	+1.0	109 239
2010	31	991	550 296	+2.4	120 676
2011	33	1314	609 973	+11.3	134 106
2012	34	1354	640 144	+4.9	143 844
2013	38	1169	686 271	+7.2	149 466
2014	39	1279	776 556	+13.1	170 163
2015	38	1343	849 811	+10.2	187 542
2016	40	1347	918 159	+8.0	195 766
2017	39	1382	940 503	+2.4	198 215
2018	39	1422	1 007 598	+7.1	215 610
**Total**			11 726 598		2 275 585

The steepest rise in treatment numbers was observed for FET (+14%; +8.2% in 2017), ED (+16.2%; –6.1% in 2017) and PGT (+29.5%; +37.8% in 2017).

**Figure 1.**
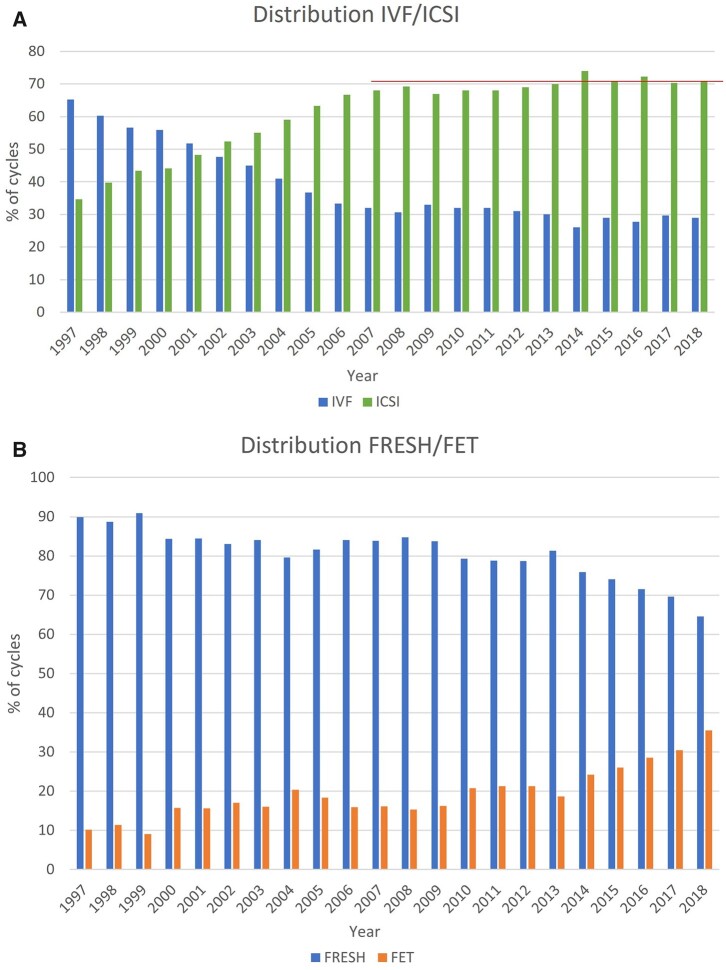
**Distribution of treatments in Europe, 1997–2018.** (**A**) Proportion of IVF versus ICSI cycles. (**B**) Proportion of fresh versus frozen cycles.

Figure 1A shows the evolution and continuing preponderance of ICSI over conventional IVF. Among a total of 563 212 fresh treatments (ICSI + IVF), 71.1% (70.3% in 2017) were done with ICSI.

The highest proportions of FET treatments (calculated as FET/(FET + ICSI + IVF)) were reached in Armenia (+55.1%), The Netherlands (49.8%), Czech Republic (48.9%), Ukraine (46.9%), Finland (46.1%), Belgium (45.8%), Albania (44.5%), Switzerland (43.3%) and Poland (43%), with an overall proportion of 35.5% and comparable to 32.6% in 2017 (Fig. 1B).

The number of cycles per million women of reproductive age and per million inhabitants is shown in Table I and Supplementary Table SIV. Availability of ART treatments was calculated for the 21 countries with full coverage (Supplementary Table SIV) showing a huge variability in availability when all techniques are considered (range per million women aged 15–45 years: 2335 in Lithuania to 19181 in Denmark); corresponding proportions of newborns resulting from ART were 0.9% and 5.7% of all newborns in these countries. Among other countries with complete reporting to the national registry, proportions of ART infants above 5% were calculated for Austria, Czech Republic, Iceland and Slovenia.

### Pregnancies and deliveries after treatment

Table III shows pregnancy and delivery rates after IVF or ICSI and after FET (after both IVF and ICSI). Outcome data were calculated per aspiration rather than per initiated cycle as the numbers of initiated cycles have often been incompletely reported.

**Table III:** Results after ART in 2018.

	IVF				ICSI			FET				
Country	Initiated cycles IVF + ICSI	Aspirations	Pregnancies per aspiration (%)	Deliveries per aspiration (%)	Aspirations	Pregnancies per aspiration (%)	Deliveries per aspiration (%)	Thawings FET	Pregnancies per thawing (%)	Deliveries per thawing (%)	ART infants^†^	ART infants per national births (%)
Albania	86				86	30.2	18.6	69	33.3	27.5	62	
Armenia	1753	783	25.3	24.1	954	18.8	17.5	2156	38.8	31.2	1412	3.4
Austria	10 828	1701	28.7	24.0	5703	27.1	22.5	3161	33.2	28.4	5372	6.3
Belarus	3292	1223	32.2	20.9	1884	29.4	17.9	992	35.9	21.0	945	1.1
Belgium	19 032	2554	21.3	15.3	13 783	21.8	15.6	13 821	27.8	19.2	5973	5.0
Bosnia-Herzegovina, Federation part	111				98	32.7	22.4	58	48.3	27.6	41	
Bulgaria	9482	1078	12.2	7.7	8404	14.7	11.3	2469	30.8	23.0		
Czech Republic	15 435	1426	7.8	6.3	13 446	22.2	13.8	14 749	30.7	18.0	6281	5.5
Denmark		6479	19.4	12.7	5536	19.2	15.4	6912	28.5	21.9	3534	5.7
Estonia	2066	638	24.0	19.0	1325	24.4	18.8	1044	29.0	19.0	702	4.9
Finland	4372	2371	22.9	18.0	1741	21.0	16.8				1791	3.7
France		20 821	19.7	16.7	41 021	19.9	17.0	41 258	24.4	20.3	21 125	2.8
Germany	75 726	19 347	25.5	18.9	50 350	25.1	18.6	29 334	27.4	19.0	21 924	
Greece	16 285	2111	20.5	14.0	14 174	13.8	8.7	5623	38.6	17.8	5022	5.8
Hungary											1734	2.0
Iceland	489	272	25.7	19.5	183	31.1	27.3	307	41.4	31.9	236	5.6
Ireland	607	239	30.1	23.8	287	33.4	24.0	408	37.5	24.0	317	0.5
Italy	51 086	7160	20.9	14.2	39 227	17.4	11.3	19 587	30.6	21.1	12 949	2.9
Kazakhstan	6510	1555	29.6	21.2	4955	18.5	15.7				3140	
Latvia	582	143	17.5	13.3	439	21.0	13.7	359	42.9	29.2	219	
Lithuania	877	472	47.2	22.2	405	37.3	14.1	201	30.3	23.9	255	0.9
Luxembourg	735	160	28.1	21.9	516	26.0	18.4				211	3.4
Malta								2			58	1.3
Moldova	1248				1055	36.5	31.3					
Montenegro	586				569	26.2	21.8	57	29.8	24.6	174	2.4
North-Macedonia	2637	332	41.3	13.3	2012	28.7	23.7	411	32.8	26.3	818	3.8
Norway	7369	3975	26.3	22.8	3056	25.6	21.6	4178	25.7	21.4		
Poland	16 484	262	27.5	21.4	15 989	22.5	13.9	12 423	36.6	23.1	6177	1.6
Portugal	6216	2351	23.5	17.6	3457	19.4	14.7	2725	33.9	23.9	2453	2.8
Romania	3999	1632	30.4	23.1	2233	27.6	17.9	2174	39.7	28.8	1581	
Russia	92 546	37 516	27.7	20.1	52 093	25.3	17.6	43 996	41.4	28.7	37 987	2.4
Serbia	150	102	31.4	18.6	48	25.0	20.8	16	37.5	37.5	46	0.1
Slovenia	3394	1158	29.8	22.5	2112	23.9	19.5	1568	33.3	24.5	1161	6.0
Spain	49 125	5925	25.0	18.4	38 706	19.9	14.4	31 894	37.5	26.3	34 541	9.3
Sweden	11 723	5611	27.1	21.9	5365	25.9	22.0				5108	4.3
Switzerland	6370	838	24.3	17.3	5027	21.1	16.1	4871	31.1	21.5	2161	2.5
The Netherlands	13 445	5541	29.9	21.3	6398	32.3	23.7					
Ukraine	12 038	598	31.4	26.9				10 620	49.5	40.6	8792	2.7
UK	40 722	17 274	31.5	27.8	20 974	31.7	27.9	22 505	35.5	31.0	21 312	2.9
**All**	487 406	153 648	25.5	19.6	36 3611	22.5	16.7	279 948	33.4	24.2	215 614	3.5

Total rates refer to these countries were all data were reported for the given technique.

† ART infants also include egg donation (ED).

For IVF and ICSI, there were for France, Greece, Ireland, Kazakhstan, Russia and Spain, respectively 177, 46, 1, 8, 543 and27 deliveries with unknown outcome. These were accepted as singletons to calculate the ART infants.

For frozen embryo transfer (FET), there were for France, Greece, Kazakhstan, Russia and Spain, respectively, 41, 4, 2, 8 and 4 deliveries with unknown outcome. These were accepted as singletons to calculate the ART infants.

For the Netherlands, no data on the number of thawings were available.

For ED, there were for France, Greece, Kazakhstan, Poland, Russia, Spain and Ukraine, respectively, 1, 2, 1, 1, 23, 8 and 9 deliveries with unknown outcome. These were accepted as singletons to calculate the ART infants.

For PGT, there was for Russia one delivery with unknown outcome. This one was accepted as singleton to calculate the ART infants.

In the Czech Republic, IVF and ICSI were reported together, no details on pregnancies and deliveries.

PGT, preimplantation genetic testing.

Among the 39 reporting countries, 36 were able to provide both pregnancy and delivery rates per aspiration after IVF (n = 33) and/or ICSI (n = 36). For FET when considering thawing cycles, 32 countries were able to report pregnancy and delivery rates (28 in 2017). Supplementary Table SIV shows the numbers of deliveries for the 21 countries with full coverage of the reporting.

Significant variation for pregnancy and delivery rates (for all types of treatment cycles) was observed between countries as in previous years.

**Figure 2.**
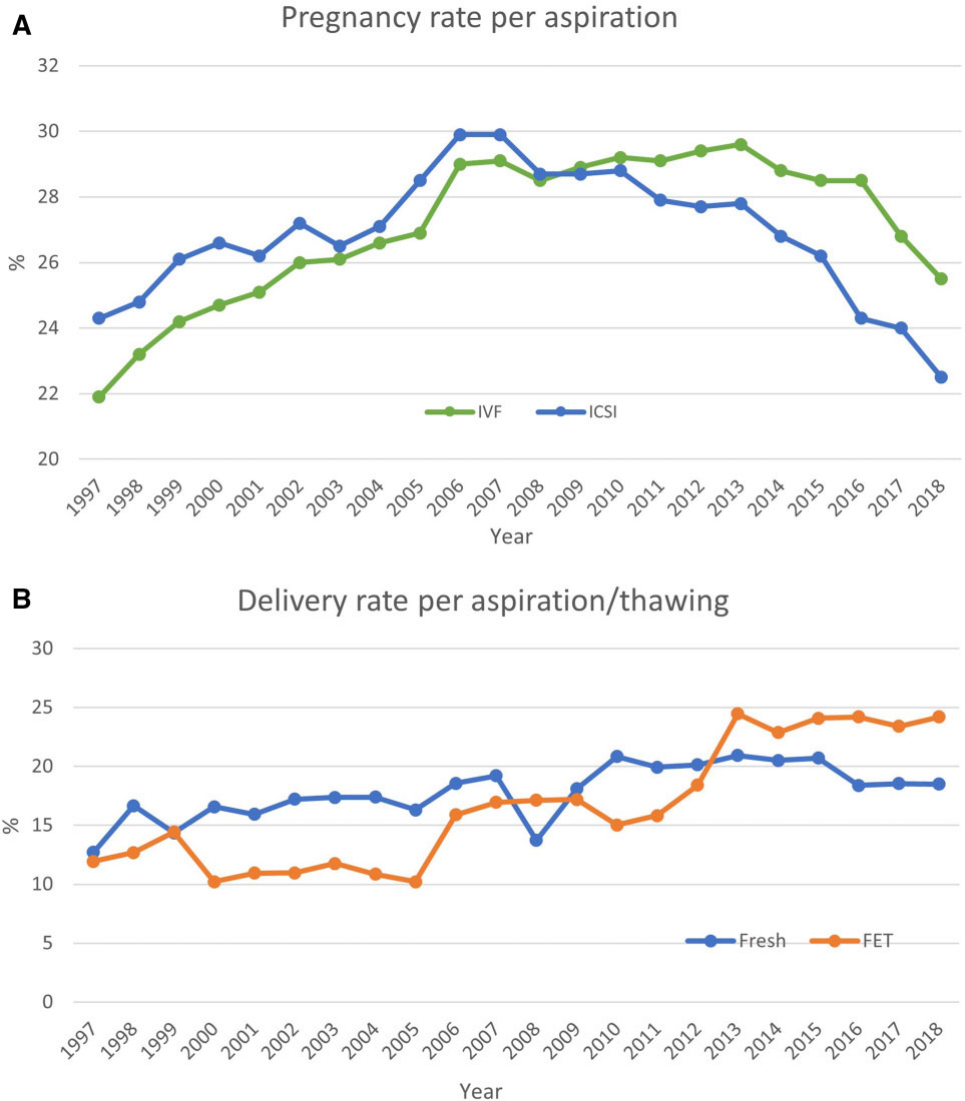
**Pregnancy and delivery rates per aspiration in Europe, 1997–2018.** (**A**) Pregnancy rates for IVF versus ICSI cycles. (**B**) Delivery rates for fresh versus frozen cycles.

**Figure 3.**
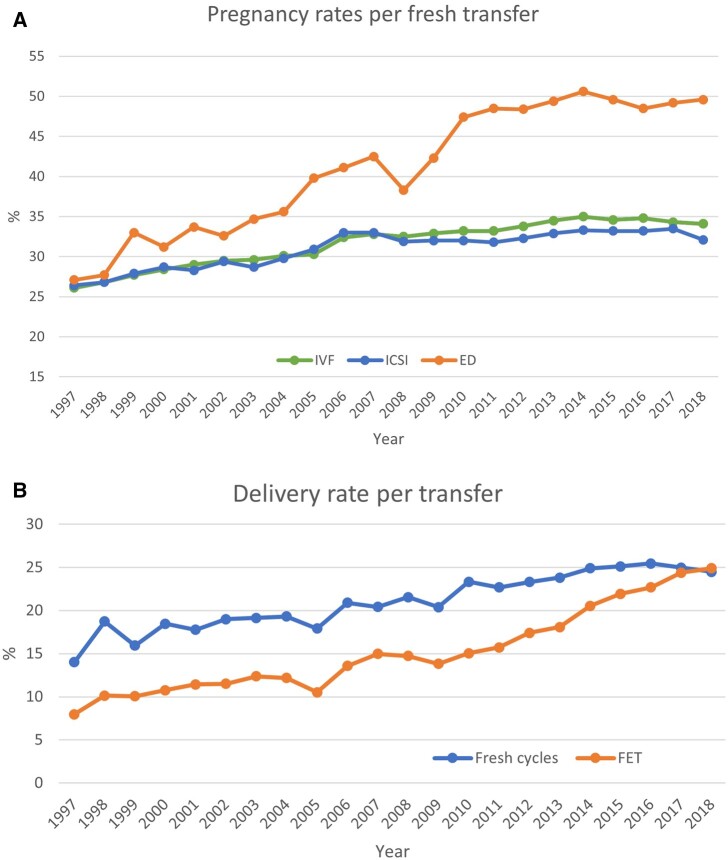
**Pregnancy and delivery rates per transfer in Europe, 1997–2018.** (**A**) Pregnancy rates for IVF versus ICSI and ED cycles. (**B**) Delivery rates for fresh versus frozen cycles. ED, egg donation.

Per aspiration, total pregnancy rates are shown in Fig. 2A and ranged from 7.8% to 47.2%. The total delivery rates per aspiration are shown in Fig. 2B and ranged from 6.3% to 31.3% in fresh cycles after IVF or ICSI (including the freeze-all cycles whether performed or not by the countries) (Table III). For FET, the pregnancy and delivery rates per thawing varied between 24.4% and 49.5% and between 17.8% and 40.6%, respectively. Overall, while higher pregnancy and delivery rates were recorded for FET cycles (per thawing) than for both fresh IVF and ICSI cycles (per aspiration) (Table III;Supplementary Table SVII), the pregnancy rates per transfer in fresh cycles remained at the same level (34.1% for IVF and 32.1% for ICSI; Fig. 3A) as in FET cycles (34.3%), as did delivery rates per transfer (26.1% for IVF, 23.9% for ICSI and 24.9% for FET), as in 2017 (Supplementary Tables SV–SVII; Fig. 3B).

When considering the stage of replaced embryos, the data showed pregnancy rates for blastocyst transfers to be higher (40.0% versus 28.5% for cleavage stage embryos for fresh IVF and ICSI cycles together, and 38.6% versus 27.2% for cleavage stage embryos for FET).

Cycle numbers, aspirations, transfers, pregnancies, deliveries in IVF, ICSI and FET (after both IVF and ICSI) by country are presented in Supplementary Tables SV–SVII.

For the fifth time, freeze-all cycles were collected (Supplementary Tables SV and SVI) including either freezing of all oocytes reported by 11 countries for IVF (10 in 2017 and 10 in 2016) and 17 countries for ICSI (17 in 2017 and 15 in 2016), or of all embryos by 23 countries for IVF (22 in 2017 and 22 in 2016) and 25 countries for ICSI (27 in 2017 and 22 in 2016). The highest proportions of freeze-all cycles per aspiration (oocytes and embryos together) were 29.8% (5.4% in 2017) and 41.7% (49.1% in 2017), respectively, for IVF and ICSI.

ED cycle numbers were available for 23 countries (21 in 2017) although 27 (26 in 2017) provided outcome data (Supplementary Table SVIII). The highest numbers of ED cycles were reported from Spain, the Czech Republic and Russia, as in 2017. The number of aspirations of donated oocytes was 36 938 (34 545 in 2017) that led to 24 148 fresh transfers (26 447 in 2017), while the replacements of frozen oocytes (FOR) were 16 130 (14 129 in 2017). The pregnancy rates per fresh embryo transfer were 49.6% (49.2% in 2017) for freshly donated oocytes and 44.9% (41.1% in 2017) for thawed oocytes. A high variability was seen between countries, ranging from 31.2% to 83.3% for fresh oocytes and from 24.8% to 57.1% for thawed oocytes, as in previous years. Overall (including also the replacements of frozen embryos), 25 760 deliveries were reported with donated eggs (21 312 in 2017 and 22 497 in 2016). Compared to cycles using own oocytes, pregnancy and delivery rates per transfer were higher for fresh (IVF and ICSI) and FET cycles together.

### Age distribution

Supplementary Tables SIX and SX showed that age distributions of women treated with IVF and ICSI varied between countries. Some countries were not able to provide age categories (eight for IVF and four for ICSI). The highest percentage of women aged 40 years and older undergoing aspiration for IVF was reported by Greece (as in 2017), whereas the highest percentage of women aged <34 years was reported by Ukraine (as in 2017). For ICSI, the highest percentage of women aged 40 years and older undergoing aspiration was also reported by Greece (as in 2017), whereas the highest percentage of women undergoing aspiration aged <34 years was recorded in Sweden (Albania in 2017). An age-dependent decrease of pregnancy and delivery rates for IVF and ICSI cycles was reported, as expected. Pregnancy and delivery rates in women aged 40 years and older ranged between 5.3% and 32.3%, and 0% and 22%, respectively. The age-related decline was also visible in FET cycles (Supplementary Table SXI) with recorded pregnancies and delivery rates among women aged 40 years and older ranging from 0 to 49.1% and 0 to 40%, respectively.

As seen in Supplementary Table SXII, the age of the recipient women had no influence on outcomes of ED cycles.

### Numbers of embryos transferred and multiple births

The number of embryos replaced per transfer procedure after IVF and ICSI together as well as multiple birth rates per subgroups defined by the number of embryos replaced are presented in Table IV.

**Table IV:** Number of embryos transferred after ART and deliveries in 2018.

	IVF + ICSI								FET		
Country	Transfers	One embryo (%)	Two embryos (%)	Three embryos (%)	Four+ embryos (%)	Deliveries	Twin (%)	Triplet (%)	Deliveries	Twin (%)	Triplet (%)
Albania	69	14.5	85.5	0.0	0.0	16	25.0	0.0	19	15.8	0.0
Armenia	936	27.8	48.4	23.8	0.0	356	9.8	0.8	673	5.5	0.4
Austria	9074	72.9	26.9	0.2	0.0	5174	3.6	0.1			
Belarus	2480	30.4	61.6	8.0	0.0	593	21.7	0.0	208	10.6	1.0
Belgium	12 135	70.4	26.1	3.2	0.3	2543	6.1	0.2	2660	5.0	0.0
Bosnia-Herzegovina, Federation part	83	54.2	41.0	4.8	0.0	22	13.6	0.0	16	0.0	0.0
Bulgaria											
Czech Republic	10 500	76.7	22.9	0.4	0.0	1942	5.3	0.1	2660	6.9	0.1
Denmark	8580	80.6	19.1	0.3	0.0	1678	2.3	0.1	1512	2.5	0.0
Estonia	1529	53.1	44.4	2.5	0.0	370	11.6	0.5	198	9.1	0.0
Finland	2855	94.3	5.7	0.0	0.0	719			878		
France	41 180	55.4	41.2	3.1	0.2	10 465	10.3	0.2	8356	6.6	0.1
Germany	54 531	30.5	65.3	4.2	0.0	13 034	19.3	0.4	5562	12.5	0.3
Greece	15 265	20.7	54.8	19.0	5.5	1529	20.3	0.5	1003	24.3	0.2
Hungary	6355	0.0	0.0	0.0	0.0	1241	19.3	0.6	208	14.4	0.5
Iceland	321	98.4	1.6	0.0	0.0	103	1.9	0.0	98	1.0	0.0
Ireland	402	71.1	28.9	0.0	0.0	129	2.3	0.0	99	5.1	1.0
Italy	30 584	39.1	49.4	10.9	0.6	5458	13.6	0.3	4127	6.7	0.1
Kazakhstan	3489	46.2	50.4	3.4	0.0	1106	16.6	0.1	1203	17.5	0.3
Latvia	313	80.5	18.5	1.0	0.0	79	2.9	0.0	105	4.4	0.0
Lithuania	701	23.5	38.8	37.8	0.0	162	24.0	4.2	48	25.0	0.0
Luxembourg	503	50.5	49.5	0.0	0.0	130	3.8	0.0	73	4.1	0.0
Malta	212	15.1	67.5	17.5	0.0	46	21.7	2.2			
Moldova											
Montenegro	423	22.7	40.9	36.4	0.0	124	27.4	0.0	14	14.3	0.0
North-Macedonia	1851	32.6	60.5	6.9	0.0	521	21.1	0.0	108	13.9	0.0
Norway											
Poland	9767	62.9	36.8	0.3	0.0	2277	8.0	0.0	2865	5.7	0.0
Portugal	3663	44.0	55.7	0.3	0.0	921	13.0	0.2	652	10.5	0.3
Romania	2478	39.3	52.1	8.1	0.5	776	13.3	0.1	626	11.9	0.0
Russia	64 968	52.3	46.9	0.7	0.0	16 688	13.7	0.3	12 606	12.8	0.2
Serbia	117	28.2	29.1	42.7	0.0	29	24.1	3.4	6	33.3	0.0
Slovenia	2610	56.8	43.1	0.2	0.0	672	9.2	0.0	384	7.0	0.0
Spain	25 693	41.7	56.7	1.7	0.0	6671	13.6	0.1	8388	10.2	0.1
Sweden	8590	87.3	12.7	0.0	0.0	2409	2.7	0.0	2392	2.3	0.0
Switzerland	3705	64.5	33.6	1.9	0.0	956	9.4	0.0	1047	6.3	0.1
The Netherlands											
Ukraine	5493	40.2	53.0	6.8	0.0	1828	16.0	0.1	4312	14.9	0.1
United Kingdom	33 119	64.5	33.3	2.1	0.0	10 660	8.1	0.1	6979	8.8	0.2
All^*^	364 574	50.7	45.1	3.9	0.3	91 427	12.4	0.2	70 085	9.4	0.1

* Totals refer only to these countries where data on number of transferred embryos and on multiplicity were reported.

**Figure 4.**
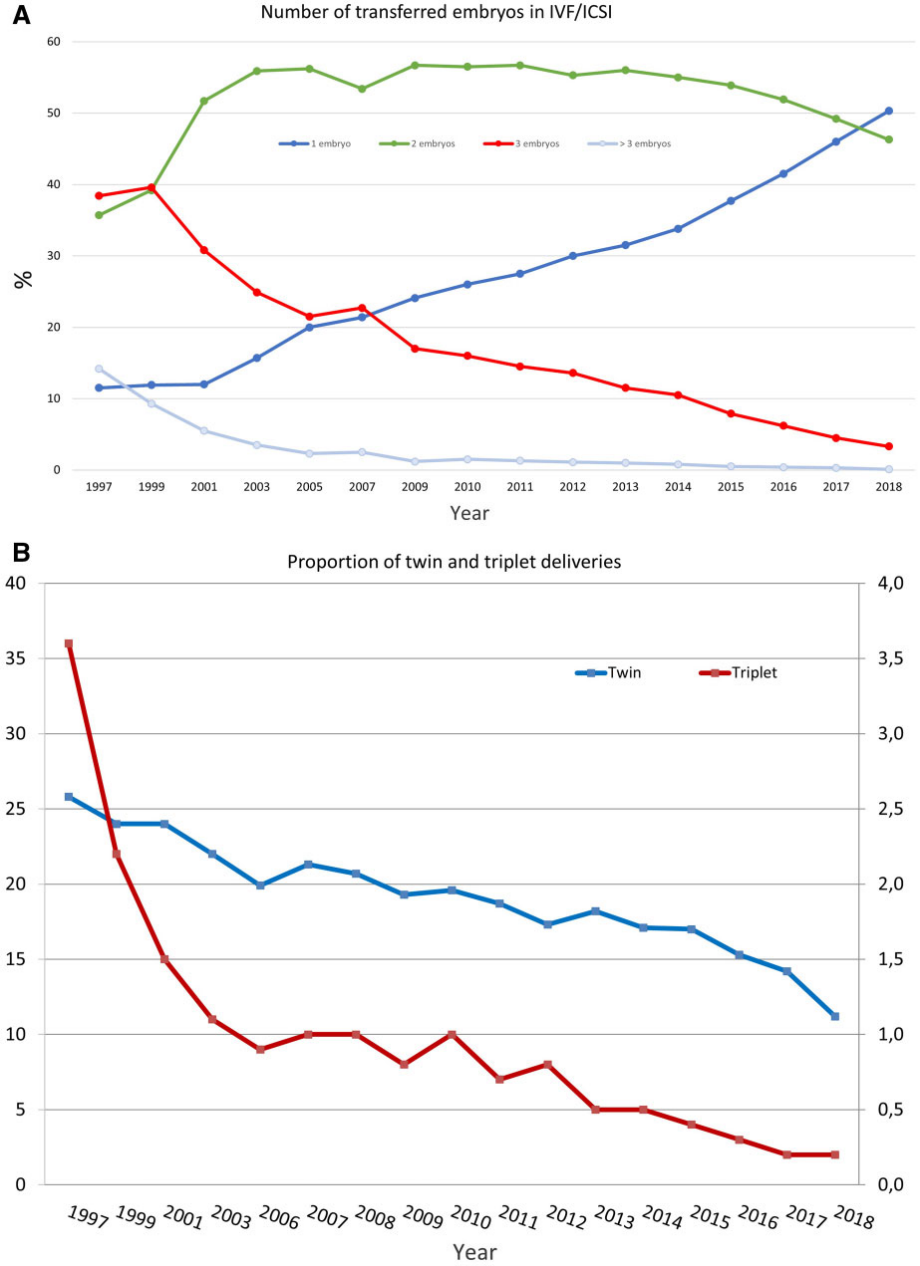
**Embryo transfer and multiple births in Europe, 1997–2018.** (**A**) Number of embryos transferred in IVF and ICSI during fresh cycles. (**B**) Percentages of twin and triplet deliveries.

Four countries did not report either on the number of replaced embryos or on multiplicity. For the first time, most transfers involved the replacement of one embryo (elective or not) (50.7% of cycles versus 46% with single embryo replacement in 2017). The evolution of the proportions of replacements of one, two and three or more embryos is shown in Fig. 4A.

Eighteen countries reported more than 50% of single embryo transfers (13 in 2017) with six reporting more than 75% of single embryo transfers. For the second year, none of the reporting countries carried out more than 50% of their transfers with three embryos. Among five countries recording transfers of four or more embryos, the highest proportion was recorded in Greece (5.5% versus 4% in 2017). For the third consecutive year, data on the embryo stage at transfer was collected. Taking into account that the embryo stage at transfer was unknown for 20.8% of the fresh (IVF + ICSI) cycles, 50.1% (44.1% in 2017) of the transfers were performed at the blastocyst stage. The corresponding figure for FET was 73.9% (64.1% in 2017). Such information was not available for each of the subgroups for numbers of embryos replaced.

As a result of decreasing numbers of embryos replaced per transfer, the global proportion of twin and triplet deliveries continued to decrease (Fig. 4B). Twin and triplet rates for fresh IVF and ICSI cycles together were 12.4% (range: 1.9–27.4) and 0.2% (range: 0–4.2), respectively: corresponding results for FET were 9.4% and 0.1%. Two countries reported rates of single embryo replacement above 90% in fresh cycles (98.4% for Iceland, 94.3% for Finland) and twin rates were as low as 1.9% (for Iceland, not available for Finland).

Supplementary Tables SXIII and SXIV provide additional information on pregnancies and deliveries. The reported incidence of pregnancy loss was 19.3% after removing countries where no pregnancy loss was documented (16.6% in 2017) after IVF + ICSI, and 21.4% (18.3% in 2017) after FET. The proportion of recorded lost to follow-up was 7.2% after IVF + ICSI (7.5% in 2017) and 7.2% after FET (8.1% in 2017).

### Perinatal risks and complications

**Figure 5.**
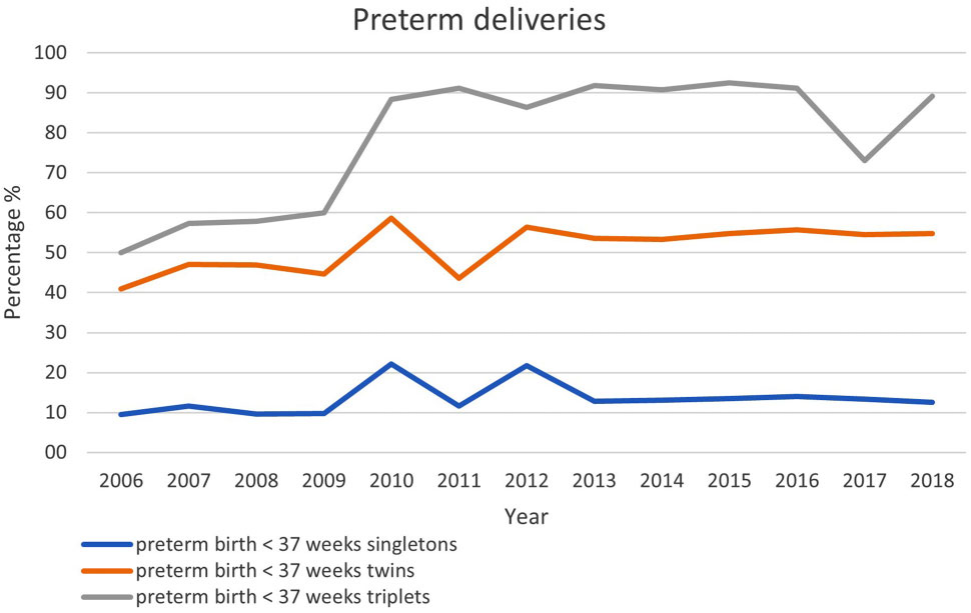
Proportion of premature deliveries (<37 weeks of gestation in relation to pregnancies ≥37 week of gestation) in singleton, in twin and in triplet pregnancies in Europe, 2006–2018.

Data on premature deliveries were available from 21 countries (19 countries in 2017). Premature delivery rates (for fresh IVF and ICSI, FET and ED together) according to multiplicity are presented in Supplementary Table SXV. The incidence of extreme preterm birth (20–27 gestational weeks at delivery) was 1% in singletons (1.1% in 2017), 3.1% in twins (3.4% in 2017) and 6% in triplets (10.7% in 2017). Very premature birth rates (28–32 gestational weeks at delivery) were recorded in 2.2% of singletons (2.4% in 2017), 9.7% of twin pregnancies (10.3% in 2017) and 37.9% in triplet pregnancies (21.7% in 2017). The evolution of the proportions of premature deliveries (before 37 weeks) over the years according to multiplicity is shown Fig. 5. Term deliveries (≥37 weeks) were achieved in 83.1% (86.6% in 2017) of singleton pregnancies, 43.6% (45.2% in 2017) of twin pregnancies and 8.1% (27% in 2017) of triplet pregnancies.

Complications related to ART procedures were reported by 34 countries (32 in 2017) and foetal reductions by 34 countries (24 in 2017); one country (Albania) only reported foetal reductions but no other complications (Supplementary Table SXVI). The main reported complication was ovarian hyperstimulation syndrome (OHSS) (Grades 3–5) with a total reported number of 1719 (1839 in 2017) corresponding to an incidence rate of 0.17% (0.20% in 2017). Other complications (1379; 1484 cases in 2017) were reported with a total incidence of 0.14% (0.16% in 2017) and bleeding being the most recorded (0.1%, identical to 2017). Three maternal deaths were reported (one in 2017). After inquiry, no details on a potential link with ART were obtained for two of these. One maternal death was caused by massive bilateral pulmonary embolism in a patient without identified risk factors. The causal link to the ART procedure itself could not be established with certainty. Foetal reductions were reported in 509 cases (599 in 2017), the majority from the UK, Belgium and Spain, as in 2017.

### Preimplantation genetic testing

Table I includes PGT for monogenic disorders and structural rearrangements (PGT-M/SR) and PGT for aneuploidies (PGT-A) activities, which were reported from 24 countries (25 in 2017). The main contributors were Spain, Russia, Ukraine and Italy. The total number of treatment cycles was 48 294 representing 7.1% of initiated IVF + ICSI and FET cycles together (37 303; 4.3% in 2017). More details on PGT/PGT-A activities can be found in the annual reports of the ESHRE PGT consortium (Van Montfoort *et al.*, 2021).

### IVM

A total of 532 treatments with IVM were reported from eight countries (378 treatments from the same eight countries in 2017) (Table I). Most IVM cycles were recorded in Belgium, as in 2017. A total of 254 transfers resulted in 49 pregnancies (19.3% per transfer) and 36 deliveries (14.2% per transfer).

### Frozen oocyte replacement

A total number of 5444 thawing cycles were reported by 22 countries (5210 from 21 countries in 2017) (Table I) with Italy and Spain being the largest contributors (1318 and 1069 cycles, respectively). Among 3993 transfers, 1178 resulted in pregnancies (29.5%; 27.5% in 2017) and 867 in deliveries (21.7%; 21% in 2017).

### IUI

Data on IUI-H or IUI-D were collected by a total of 1271 institutions (1273 in 2017) in 30 and 25 countries, respectively, as in 2017 (Table V). Amongst 148 143 IUI-H (155 794 in 2017) and 50 609 IUI-D (51 402 in 2017) reported cycles, the numbers were the highest for IUI-H in France, Spain, Italy and Belgium, and for IUI-D in Spain, Belgium, Denmark and the UK (Supplementary Tables SXVII and SXVIII).

**Table V:** IUI with husband (IUI-H) or donor (IUI-D) semen in 2018.

	IUI-H						IUI-D					
Country	Cycles	Deliveries	Deliveries (%)	Singleton (%)	Twin (%)	Triplet (%)	Cycles	Deliveries	Deliveries (%)	Singleton (%)	Twin (%)	Triplet (%)
Albania	61	7	11.5	57.1	42.9	0.0						
Armenia	906	124	13.7	82.3	17.7	0.0	108	17	15.7	100.0	0.0	0.0
Austria												
Belarus	1098	115	10.5	92.2	7.8	0.0	45	8	17.8	100.0	0.0	0.0
Belgium	12 382	880	7.1	95.8	4.0	0.2	9296	1026	11.0	96.9	3.1	0.0
Bosnia-Herzegovina, Federation part	130	11	8.5	100.0	0.0	0.0						
Bulgaria												
Czech Republic												
Denmark	10 108	1030	10.2	90.4	9.6	0.0	8453	556	6.6	95.7	4.3	0.0
Estonia	140	9	6.4	100.0	0.0	0.0	170	26	15.3	100.0	0.0	0.0
Finland	2570	211	8.2				968	133	13.7			
France	44 390	4568	10.3	91.3	8.4	0.3	2789	526	18.9	91.8	8.0	0.2
Germany												
Greece	2715	213	7.8	96.2	3.8	0.0	373	65	17.4	93.8	6.2	0.0
Hungary	2786	158	5.7	90.5	9.5	0.0						
Iceland	19	6	31.6	100.0	0.0	0.0	182	32	17.6	100.0	0.0	0.0
Ireland	141	14	9.9	92.3	0.0	7.7	90	11	12.2	100.0	0.0	0.0
Italy	17 083	1276	7.5	91.1	8.4	0.5	667	93	13.9	86.0	11.8	2.2
Kazakhstan	485	50	10.3	80.0	18.0	2.0	51	16	31.4	50.0	50.0	0.0
Latvia	63	7	11.1	71.4	28.6	0.0	50	4	8.0	100.0	0.0	0.0
Lithuania	382	29	7.6	92.3	7.7	0.0	7	2	28.6	100.0	0.0	0.0
Luxembourg	249	24	9.6	83.3	16.7	0.0	78	13	16.7	76.9	23.1	0.0
Malta												
Moldova	87											
Montenegro	202	25	12.4	96.0	4.0	0.0						
North-Macedonia	796	26	3.3	100.0	0.0	0.0	31	1	3.2	100.0	0.0	0.0
Norway	276	31	11.2	86.7	13.3	0.0	815	109	13.4	95.4	4.6	0.0
Poland	11 918	700	5.9	94.6	5.3	0.1	1863	222	11.9	94.8	4.7	0.5
Portugal	2137	180	8.4	90.6	8.9	0.6	426	72	16.9	87.5	12.5	0.0
Romania	1625	90	5.5	93.3	6.7	0.0	144	18	12.5	77.8	22.2	0.0
Russia	8486	861	10.1	92.5	6.6	0.8	3279	413	12.6	95.4	4.6	0.0
Serbia	432	28	6.5	92.9	3.6	3.6						
Slovenia	594	53	8.9	86.8	11.3	1.9						
Spain	21 467	2183	10.2	88.9	10.9	0.2	12633	1837	14.5	89.7	10.0	0.3
Sweden							2120	329	15.5	98.2	1.8	0.0
Switzerland												
The Netherlands												
Ukraine	1056	75	7.1	93.3	6.7	0.0	315	52	16.5	96.2	3.8	0.0
UK	3359						5656	798	14.1	94.9	4.6	0.5
All^*^	148 143	12984	8.9	91.2	8.4	0.3	50609	6379	12.6	93.4	6.4	0.2

* Total refers to these countries were data were reported and mean percentage were computed on countries with complete information.

Italy, Spain: underestimation of deliveries because of high number of pregnancies is lost to follow up.

Macedonia: data from two clinics only.

Poland: for IUI with husband sperm (IUI-H) and IUI with donor sperm (IUI-D), there were, respectively, 282 and 63 pregnancies with unknown outcome.

Delivery rates could be calculated for 144 697 IUI-H cycles (8.9%; 8.9% in 2017) and 50 609 for IUI-D cycles (12.6% versus 12.4% in 2017). Singleton deliveries were the most frequent regardless of the age group with an overall rate of 91.2% for IUI-H and 93.4% for IUI-D (91.6% in IUI-H, 92.9% in IUI-D in 2017). Twin and triplet rates were 8.4% and 0.3% for IUI-H, and 6.4% and 0.2% for IUI-D, respectively (in 2017: 8.1% and 0.3% for IUI-H and 6.9% and 0.2% respectively for IUI-D).

### Sum of fresh and FET (‘cumulative’) delivery rates

Supplementary Table SXIX provides an estimate of a cumulative delivery rate. It was calculated as the ratio between the total number of deliveries from fresh embryo transfers and FET performed during a year (numerator) and the number of aspirations during the same year (denominator) and is thus different from a true cumulative delivery rate, which is based on all transfers resulting from one aspiration. The calculation was based on data from 36 countries (34 countries in 2017), where an overall rate of 32.3% (30.8% in 2017) was recorded. The cumulative increase resulting from additional FET (over delivery rates from fresh embryo transfers) was 14.4% (12.3% in 2017), with the highest benefits reported by Armenia (+38.7%), Albania (+22.1%), Sweden (+21.8%), Iceland (+21.6%) and Finland (+21.3%), and the lowest reported by Montenegro (+2.5%), Serbia (4%), North-Macedonia (+4.6%) and Moldova (+4.7%).

### Cross-border reproductive care

Twelve countries reported data on cross-border reproductive care: Albania, Belarus, Bosnia-Herzegovina (Federation Part), Denmark, Greece, Iceland, Lithuania, Poland, Portugal, Slovenia, Spain and Switzerland. A total of 21 792 cycles (16 733 in 2017) were reported, 21.5% (16.9% in 2017) of which involved IVF/ICSI with the couple’s own gametes, 52.6% (50.1% in 2017) were oocyte donations and 20.6% (25.8% in 2017) were IVF or ICSI with semen donation. Additionally, 6791 IUI with sperm donation (7298 in 2017) were registered. Information regarding the countries of origin was very incomplete and not reliable enough to obtain any conclusive information. The main reason reported by patients for crossing the borders was to seek a higher quality treatment (42.3%; 25.7% in 2017). However, cross-border reproductive care was also reported to be performed because the treatment was not legal (21.1%; 41.9% in 2017) or too expensive in the home country (25%; 19% in 2017), or because the waiting list was too long (11.5%; 8% in 2017).

### Fertility preservation

For the third year, data on FP were reported. Sixteen countries (14 in 2017 and 11 in 2016) provided data on a total number of 20 994 interventions (18 888 in 2017; 13 689 in 2016) (Supplementary Table SXX) both for medical and non-medical reasons in pre- and post-pubertal patients. The majority of interventions consisted of the cryopreservation of ejaculated sperm (n = 10 503 from 14 countries; 11 112 from 13 countries in 2017) and the cryopreservation of oocytes (n = 9123 from 16 countries; 6588 from 13 countries in 2017). Ovarian tissue cryopreservation was reported by 2 (3 in 2017) and 11 (10 in 2017) countries, respectively, for pre- and post-pubertal patients with use of post-pubertal tissue through transplantation reported in four countries (Greece, Italy, Poland and Spain). Testicular tissue cryopreservation in post-pubertal patients and pre-pubertal boys was reported from eight (8 in 2017) countries and from one country (4 in 2017), respectively.

## Discussion

From 1997 to 2018, the EIM Consortium of ESHRE has registered more than 11 million treatments cycles (11 726 598) that have led to the birth of over 2 million infants.

This 22nd annual report summarizes and analyses data on ART, IUI and FP activities collected from European compulsory or voluntary registries of 39 European countries (as in 2017). For the first time, more than 1 million treatment cycles in 1 year have been reported. Only a few countries did not participate (5 of 44 EIM members, 7 non-EIM members including Azerbaijan, Kosovo and 5 countries not offering ART services). Only three member states of the EU were not able to deliver data (Croatia, Cyprus and Slovakia). Based on a survey on medically assisted reproduction (MAR) activities (Calhaz-Jorge *et al.*, 2020), the most likely reasons for not being able to send data are either economic at the centre and/or country level, regulatory or political.

Overall, while the number of European countries participating has remained quite stable over the last few years, with only slight fluctuations in some countries, the reported treatment cycle numbers continue to rise (+7.1% as compared to 2017), as do the number of infants born from ART (+8.8% as compared to 2017).

Awareness of the crucial role of registries on MAR activities is growing fast. Indeed, efforts of EIM and the collaboration of the EU affairs committee of ESHRE with the EU Directorate General for Health and Food Safety (DG SANTE), led to a broad reflection on the future of registries in the field of reproductive care; knowing that they are key to improving clinical care based on both outcome parameters and safety in MAR treatments (De Geyter *et al.*, 2016; Kissin *et al.*, 2019). Registries in MAR should aim at reaching the highest level of completeness and harmonization of European data, and joined competences should lead to a flexible common IT solution for data collection from all participating countries. EIM data are also included in the annual report of the world IVF register of ICMART (Chambers *et al.*, 2021).

Despite well-known challenges linked to heterogeneous systems and lack of harmonization of indicators, the participation rate at the country level is as high as 86.3% of all European countries (88.6% of EIM members) after exclusion of those countries where ART is not provided. However, only 21 countries (47.7% of EIM members as in 2017) were able to send data from all institutions offering IVF services, resulting in a proportion of 91.6% of all IVF institutions sending in their data (versus 90.1% in 2017; Wyns *et al.*, 2021). Therefore, next efforts should first focus on reaching the collection of complete data sets within countries.

Further progress towards a higher quality of the data from participating countries is expected through achieving a prospective cycle-by-cycle data collection (ongoing for 14 countries in 2018) with harmonized indicators. In this regard, as a first step, a minimum core data set on outcome parameters with definitions of collected items was established (https://www.eshre.eu/Data-collection-and-research/Consortia/EIM). Until higher quality data, including harmonization of collection systems among countries and registration of indicators taking into account centre/country-specific practices (e.g. freeze-all cycles, embryo transfer policy, PGT-A, etc.), become available, the interpretation of the data should remain cautious.

Besides the current EU objective of installing better vigilance in MAR, increased transparency on access to reproductive care and cross-border treatments for all stakeholders is also of importance. Over the years, EIM has constantly recorded a high variability in access to treatment between countries, ranging per million women aged 15–45 years from 2335 in Lithuania to 19 181 in Denmark, and per million inhabitants from 389 in Lithuania to 3549 in Denmark.

While such data are unique in Europe, interpretation becomes more difficult as the historical estimated threshold of 1500 fresh ART cycles needed for infertility care per million inhabitants is becoming obsolete owing to numerous technological developments in the field. Cross-border patients also matter when best estimates of thresholds for sufficiency need to be established. Unfortunately, data on cross-border care were available for only 12 countries in 2018 (8 in 2017).

Concerning treatment modalities, ICSI remains the most applied with a trend to stabilization of its use during the last years (Table I;Fig. 1). FET is the second most used technique. Over the years, higher proportions of FET treatment cycles (FET/(FET + ICSI + IVF)) (32.6% in 2017 and 35.5% in 2018) were recorded. However, the proportion of FET cycles varies considerably among countries with complete data sets (ranging from 0.8% to 99.2%) reflecting the high variability in practices. This is also observed for freeze-all cycles (Supplementary Tables SV and SVI) that have been increasingly reported since 2014 and which reach proportions per aspiration (oocytes and embryos together) of up to 29.8% (5.4% in 2017) and 41.7% (49.1% in 2017), respectively for IVF and ICSI.

Such variability should be considered when interpreting data, especially regarding the evolution of pregnancy and delivery rates for fresh IVF and ICSI cycles (per aspiration) and for FET cycles (per thawing) over the years (Table III;Figs 2A and B and 3A and B). Indeed, the higher success rates recorded for FET (per thawing) compared to fresh IVF and ICSI (per aspiration), although kept here for comparison with previous reports, is misleading and different factors that may influence outcomes should not be neglected. First, patients who benefit from embryo cryopreservation may have a better prognosis. Second, inequalities in denominators are also of importance as the average proportion of aspirations that will result in embryo transfer is most likely different from the proportion of thawings that will lead to an embryo replacement. It is also of note that recorded delivery rates per transfer were lower for FET than for both IVF and ICSI cycles. Such observations in large data collection sets are key to pinpoint research questions towards potential causes, among others the influence of hormonal support on miscarriage rates in FET cycles.

Cumulative delivery rates per cycle or per aspiration are better outcome indicators to assess treatment effectiveness (De Neubourg *et al.*, 2016) but, so far, true cumulative delivery and livebirth rates cannot be calculated by the EIM consortium as only aggregated data are collected. The addition of outcomes of fresh and FET during the same calendar year is therefore used as a proxy-indicator until a European cycle-by-cycle registry is established. When including data from 36 countries (34 countries in 2017) a ‘cumulative’ delivery rate during the 1-year period of 32.3% (30.8% in 2017) was recorded. Gains taken from additional FET (over delivery rates from fresh embryo transfers) were also highly variable, ranging from 2.5% to 38.7%, reflecting most likely differences in freezing policies and indications.

Trends are important to inform the field on uptake of data driven from registries and analyse subsequent modification of practices (Ferraretti *et al.*, 2017; De Geyter *et al.*, 2020b). For instance, dissemination of EIM data sets increased awareness among professionals on the benefit of reducing the number of embryos replaced per transfer (Fig. 4A) to diminish multiple births (Fig. 4B). As a result, for the first time, most transfers involved the replacement of one embryo (elective or not) (50.7% of cycles versus 46% with single embryo replacement in 2017). In parallel to the evolution of the number of replaced embryos, the proportion of both twin and triplet deliveries continued to decrease (Fig. 4B) with twin and triplet rates for fresh IVF and ICSI cycles together being 12.4% (range: 1.9–27.4) and 0.2% (range: 0–4.2), respectively.

In the future, it is expected that efforts will lead to the ultimate goal of the birth of one healthy child (Land and Evers, 2003) per embryo transfer and thus to the concomitant reduction of prematurity associated with multiple births (Fig. 5).

Aiming at promoting singleton pregnancies by elective single embryo transfer but also at a reduced time to pregnancy, embryo culture is often prolonged up to the blastocyst stage. However, while the benefit of blastocyst stage transfers on ART outcomes is still a matter of debate (Glujovsky *et al.*, 2016; Practice Committee of the American Society for Reproductive Medicine and Practice Committee of the Society for Assisted Reproductive Technology, 2018), data showed pregnancy rates for blastocyst transfers to be higher than for cleavage stage embryos (40.0% versus 28.5% for fresh IVF and ICSI cycles together, and 38.6% versus 27.2% for FET). However, one should remember that while blastocyst transfer results in higher pregnancy and live birth rates per transfer, it also results in lower numbers of embryos available for transfer, pointing to the importance of true cumulative outcome parameters. Unfortunately, such data that would also allow the assessment of the time to pregnancy were not available.

Besides multiplicity and prematurity, other safety aspects of ART most likely remain underreported, with the highest rate of complications for OHSS (0.17%, similar to 0.2% in 2017) and an incidence of all other complications being registered at 0.14% (0.16% in 2017). Reports on maternal deaths related to ART are even scarcer, with a reported best estimate of six maternal deaths directly related to IVF per 100 000 IVF treatments in a national cohort from The Netherlands, where OHSS and sepsis were the major causes (Braat *et al.*, 2010). It is noticeable that three maternal deaths after ART were registered in 2018 (Supplementary Table SXVI) although the link with the ART procedure was either not communicated or not established with certainty.

Furthermore, although the age of recipients in ED cycles did not modify the outcome of the cycle, risks associated with pregnancies in aging women should not be neglected as a potential additional safety aspect of the treatment. Indeed, a survey on the legislation and reimbursement aspects has shown that some countries do not have age limitations for recipients in ED cycles (Calhaz-Jorge *et al.* 2020).

Allowing for reliable comparisons of practices and identifying the safest and most efficient care should be the objective of MAR registries. This will only be obtained by upscaling the quality of collected data towards complete and harmonized data throughout Europe. Besides the establishment of clear definitions of registered items, providing the countries and competent authorities with an adapted IT solution should be the next priority. Future developments in MAR registries could be guided and enforced by new requirements established at the EU level. Collaborative work between the EU, national competent authorities and EIM experts will be of the utmost importance to eventually respond to expectations in terms of vigilance, quality of care and transparency in ART. The same new collaborative methodology would be extended to non-EU countries.

## Data availability

All data are incorporated into the article and its online supplementary material.

## Authors’ roles

C.W. drafted the manuscript and was responsible for final editing of the manuscript. C.D.G., C.C.-J., M.S.K., T.M., J.S., C.B., A.T.-S. and I.A.R. edited the manuscript. V.G. was responsible for the data collection and edited the manuscript. V.G. was responsible for raw data curation, contributed to the tables, contributed to the figures and edited the manuscript. All authors revised and approved the final manuscript.

## Funding

The study has received no external funding and all costs are covered by ESHRE.

## Conflict of interest

There are no competing interests.

## Supplementary Material

hoac022_Supplementary_Data

hoac022_Supplementary_Table_SI

hoac022_Supplementary_Table_SII

hoac022_Supplementary_Table_SIII

hoac022_Supplementary_Table_SIV

hoac022_Supplementary_Table_SV

hoac022_Supplementary_Table_SVI

hoac022_Supplementary_Table_SVII

hoac022_Supplementary_Table_SVIII

hoac022_Supplementary_Table_SIX

hoac022_Supplementary_Table_SX

hoac022_Supplementary_Table_SXI

hoac022_Supplementary_Table_SXII

hoac022_Supplementary_Table_SXIII

hoac022_Supplementary_Table_SXIV

hoac022_Supplementary_Table_SXV

hoac022_Supplementary_Table_SXVI

hoac022_Supplementary_Table_SXVII

hoac022_Supplementary_Table_SXVIII

hoac022_Supplementary_Table_SXIX

hoac022_Supplementary_Table_SXX

## Appendix

Contact persons who are collaborators and represent the data collection programmes in participating European countries, 2018.

**Albania**

Prof Orion Gliozheni, University Hospital for Obstetrics & Gynecology, Department of Obstetrics & Gynecology, Bul.B.Curri, Tirana, Albania. Tel: +355 4 222 36 32; Fax: +355 4 2257 688; Mobile: +355 68 20 29 313; E-mail: glorion@abcom.al

**Armenia**

Mr Eduard Hambartsoumian, Fertility Center, IVF Unit, 4 Tigvan Nets, 375010 Yerevan, Armenia. Tel: +374 10 544368; E-mail: Hambartsoumian@hotmail.com

**Austria**

Prof Dr Heinz Strohmer, Dr Obruca & Dr Strohmer Partnerschaft Goldenes Kreuz, Kinderwunschzentrum, Lazarettgasse 16-18, 1090 Wien, Austria. Tel: +43 401 111 400; Fax: +43 401 111 401; E-mail: heinz.strohmer@kinderwunschzentrum.at

**Belarus**

Dr Elena Petrovskaya (Alena Piatrouskaya), ART Centre ‘Embryo’, Filimonova 53, 220053 Minsk, Belarus. Tel: +375 293 830 570; E-mail: elenaembryoby@gmail.com

Dr Oleg Tishkevich, Centre For Assisted Reproduction ‘Embryo’ Belivpul, Filimonova Str. 53, 220114 Minsk, Belarus. Tel: +375 296 222 722; Fax: +375 172 376 404; Mobile: +375 296 222 722; E-mail: tishol@tut.by

**Belgium**

Prof Dr Diane de Neubourg, Antwerp University Hospital—UZA, Center for Reproductive Medicine, Drie Eikenstraat 655, 2650 Edegem, Belgium. Tel: +32 3 821 45 98; Mobile: +32 475 69 91 18; E-mail: diane.deneubourg@uza.be

Dr Kris Bogaerts, I-Biostat, Kapucijnenvoer 35 bus 7001, 3000 Leuven, Belgium. Tel: +32 (0) 16 33 68 90; Fax: +32 (0) 16 33 70 15. E-mail: Kris.Bogaerts@med.kuleuven.be

**Bosnia**

Prof Dr Devleta Balic, Zavod za humanu reprodukciju ‘Dr Balic’, Kojsino 25, 75000 Tuzla, Bosnia—Herzegovina. Tel: +387 35 260 650; Mobile: +387 611 402 22; E-mail drbalic@bih.net.ba

Prof Dr Sanja Sibincic, Health Center Medico-S, Jevrejska 58/A, 78000 Banja Luka, Bosnia—Herzegovina. Tel: +387 512 321 00; Mobile: +387 655 159 42; E-mail: sanjasibincic@gmail.com

**Bulgaria**

Irena Antonova, ESHRE certified clinical embryologist (2011), Ob/Gyn Hospital Dr Shechterev, 25-31, Hristo Blagoev Strasse, 1330 Sofia, Bulgaria. Tel: +359 887 127 651; E-mail: irendreaming@gmail.com

**Croatia**

Prof Dr Hrvoje Vrcic, Zagreb University Medical School, Obstetrics and Gynecology, Petrova 13, 10000 Zagreb, Croatia. Tel: +385 146 046 46; Fax: +385 146 335 12; E-mail: Hrvoje.vrcic@hilarus.hr

Dr Dejan Ljiljak, Clinical Hospital Center ‘Sestre milosrd’, Department for Biology of Human Reproduction, Ob/Gyn Clinic, Vinogradska c. 29, 10000 Zagreb, Croatia. Tel: +385 378 75 97; Fax: +385 137 682 72; Mobile: +385 3787 125; E-mail: dejan.ljiljak@kbcsm.hr

**Czech Republic**

Dr Karel Rezabek, Medical Faculty, University Hospital, CAR-Assisted Reproduction Center, Gyn/Ob Department, Apolinarska 18, 12000 Prague, Czech Republic. Tel: +420 224 967 479; Fax: +420 224 922 545; Mobile: +420 724 685 276; E-mail: rezabek.ivf@seznam.cz

Mgr Jitka Markova, Institute of Health Information and Statistics of the Czech Republic, Palackeho namesti 4, 12801 Prague, Czech Republic. Tel: +420 224 972 832; Mobile: +420 721 827 532; E-mail: jitka.markova@uzis.cz

**Denmark**

Dr John Kirk, Maigaard Fertilitetsklinik, Jens Baggensensvej 88 h, 8200 Arhus, Denmark. Tel: +45 86101388, Mobile: +45 28696982; E-mail: john.kirk@dadlnet.dk

**Estonia**

Dr Deniss Sõritsa, Tartu University Hospital and Elitre Clinic, Tartu, Estonia. Tel: +372 740 9930; Fax: +372 740 9931; E-mail: soritsa@hotmail.com

**Finland**

Prof Mika Gissler, THL National Institute for Health and Welfare, P.O. Box 30, 00271 Helsinki, Finland. Tel: +385 29 524 7279; E-mail: mika.gissler@thl.fi

Dr Sari Pelkonen, Department of Obstetrics and Gynaecology, Oulu University Hospital, P.O. Box 23, 90029 Oys, Finland. Tel:+358 8 3153040; E-mail: sari.pelkonen@fimnet.fi

**France**

Prof Jacques de Mouzon, 15-29 rue Guilleminot, 75014 Paris, France. Tel: +33 143 224 679; Mobile: +33 662 062 274; E-mail: jacques.de.mouzon@gmail.com

**Germany**

Dr Andreas Tandler—Schneider; Fertility Center Berlin; Spandauer damm 130; 14050 Berlin; Germany. Tel: +49 30 233 20 81 10; Fax: +49 30 233 20 81 19; E-mail: tandler-schneider@fertilitycenter-berlin.de

**Greece**

Prof Nikos Vrachnis, National Authority of Medically Assisted Reproduction, Ploutarxou 3, P.O. 10675 Athens, Greece. Tel: +30 6974441144; E-mail: nvrachnis@hotmail.com

**Hungary**

Prof Janos Urbancsek, 1st Department of Ob/Gyn, Semmelweis University, Baross utca 27, 1088 Budapest, Hungary. Tel: +36 1 266 01 15; Fax: +36 1 266 01 15; E-mail: urbjan@noi1.sote.hu

Prof G. Kosztolanyi, Department of Medical Genetics and Child Development, University of Pecs, Jozsef A.u; 7., 7623 Pecs, Hungary. Tel: +36 7 2535977; Fax: +36 7 2535972; E-mail: gyorgy.kosztolanyi@aok.pte.hu

**Iceland**

Mr Hilmar Bjorgvinsson, IVF Klinikin Reykjavik, Alfheimum 74, 104 Reykjavik, Iceland. Tel: +354 430 4000; Fax: +354 430 4040; E-mail: Hilmar.bjorgvinsson@ivfklinikin.is

**Ireland**

Prof Mary Wingfield, Merrion Fertility Clinic, National Maternity Hospital, 60 Lower Mount Street, D02NH93 Dublin, Ireland. Tel: +353 166 350 00; Mobile: +353 872 258 556; E-mail: mwingfield@merrionfertility.ie

Ms Joyce Leyden, Merrion Fertility Clinic, National Maternity Hospital, 60 Lower Mount Street, D02NH93 Dublin, Ireland. Tel: +353 166 350 00; Mobile: +353 859 290 573; E-mail: jleyden@merrionfertility.ie

**Italy**

Dr Giulia Scaravelli, Istituto Superiore di Sanità, Registro Nazionale della Procreazione Medicalmente Assistita, CNESPS, Viale Regina Elena, 299, 00161 Roma, Italy. Tel: +3906 499 04 050; Fax: +39064 99 04 324; E-mail: giulia.scaravelli@iss.it

Dr Roberto de Luca, Istituto Superiore di Sanità, Registro Nazionale della Procreazione Medicalmente Assistita, CNESPS, Viale Regina Elena, 299, 00161 Roma, Italy. Tel.: +3906 499 04 320; E-mail: roberto.deluca@iss.it

**Kazakhstan**

Prof Dr Vyacheslav Lokshin, International Clinical Center for Reproductology ‘Persona’, Utepova Street 32a, 00506 Almaty, Kazakhstan. Tel: +7 727 382 7777; Mobile: +7 701 755 8209; E-mail: v_lokshin@persona-ivf.kz

Dr Sholpan Karibayeva, International Clinical Center for Reproductology ‘Persona’, Utepova Street 32a, 00506 Almaty, Kazakhstan. Tel: +7 727 382 7777; E-mail: sh.karibaeva@gmail.com

**Latvia**

Dr Valeria Magomedova, Jusu Arsti Private Clinic, Apuzes 14, 1046 Riga, Latvia. Tel: +371 678 700 29; Fax: +371 678 704 29; E-mail: godunova@inbox.lv

**Lithuania**

Raminta Bausyte, Vilnius University Hospital Santaros Clinics, Santaros Fertility Center, Simono Staneviciaus 64-69, 07113 Vilnius, Lithuania. Tel: +370 620 86826; E-mail: raminta.bausyte@gmail.com

Ieva Masliukaite, Academic Medical Center, Center for Reproductive Medicine, Ijburglaan 1086ZJ Amsterdam, The Netherlands. Tel: +31 653 688 815; E-mail: i.masliukaite@amc.uva.nl

**Luxembourg**

Dr Caroline Schilling, Centre Hospitalier de Luxembourg, Centre de Stérilité et de Médecine de Reproduction, Rue Fiederspiel 2, 1512 Luxembourg, Luxembourg. Tel: +352 44 11 32 30; Mobile: +352 66 13 13 912; E-mail: schilling.caroline@chl.lu

**Malta**

Dr Jean Calleja-Agius, University of Malta, 12, Mon Nid, Gianni Faure Street, TXN2421 Tarxien, Malta. Tel: +356 216 930 41; Mobile: +356 995 536 53; E-mail: jean.calleja-agius@um.edu.mt

**Moldova**

Prof Dr Veaceslav Moshin, Medical Director at Repromed Moldova, Center of Mother @ Child Protection, State Medical and Pharmaceutical University ‘N.Testemitanu’, Bd. Cuza Voda 29/1, Chisinau, Republic of Moldova. Tel: +37322 263855; Mobile: +37369724433; E-mail: mosin@repromed.md; veaceslavmoshin@yahoo.com

**Montenegro**

Dr Tatjana Motrenko Simic, Human Reproduction Center Budva, Prvomajska 4, 85310 Budva, Montenegro. Tel: +382 33402432; Mobile: +382 69 052 331; E-mail: motrenko@t-com.me

Dragana Vukicevic, Hospital ‘Danilo I’, Humana reprodukcija, Vuka Micunovica bb, 86000 Cetinje, Montenegro. Tel: +382 675 513 71; E-mail: vukicevic.dragana@yahoo.com

**The Netherlands**

Dr Jesper M.J. Smeenk, Department of Obstetrics and Gynaecology, St. Elisabeth Hospital Tilburg, Hilv, The Netherlands. Tel: +31 13 539 31 08; Mobile: +31 622 753 853; E-mail: j.smeenk@elisabeth.nl

**North Macedonia**

Mr Zoranco Petanovski, Hospital ReMedika, Nas. Zelezara, 1000 Skopje, Macedonia. Tel: +389 224 475 45, Fax: +389 226 031 00; E-mail: zpetanovski@yahoo.com

**Norway**

Dr Liv Bente Romundstad, Spiren Fertility Clinic, Nardoskrenten 11, 7032 Trondheim, Norway. Tel: +47 73523000; Mobile: +47 90550207; E-mail: libero@klinikkspiren.no

**Poland**

Dr Anna Janicka, VitroLive, Wojska Polskiego 103, 70-483 Szczecin, Poland. Tel: +48 91 88 69 260; E-mail anna.janicka@vitrolive.pl

**Portugal**

Prof Dr Carlos Calhaz-Jorge, CNPMA, Assembleia da Republica, Palacio de Sao Bento, 1249-068 Lisboa, Portugal. Tel: +351 21 391 93 03; Fax: +351 21 391 75 02; E-mail: calhazjorgec@gmail.com

Ms Joana Maria Mesquita Guimaraes. Hospital Geral Santo Antonio, Largo Professor Abel Salazar, 4050-011 Porto, Portugal. Tel: +351 96 616 02 37; E-mail: joanamesquitaguimaraes@gmail.com

Ms Ana Rita Laranjeira, CNPMA, Assembleia da Republica, Palaio de Sao Bento 1249-068 Lisboa, Portugal. Tel: +351 21 391 93 03; Fax: +351 21 391 75 02; E-mail: cnpma.correio@ar.parlamento.pt

**Romania**

Mrs Ioana Rugescu, Gen Secretary of AER Embryologist Association and Representative for Human Reproduction Romanian Society, Romania. Tel: +40744500267; E-mail: irugescu@rdsmail.ro

Dr Bogdan Doroftei, University of Medicine and Pharmacy Iasi; Teaching Hospital Obgyn ‘Cuza Voda’; Cuza Voda Str. 34, 700038 Iasi, Romania. Tel: +40 232 213 000/int. 176; Mobile: +40 744 515 297; E-mail bogdandoroftei@gmail.com; bogdan.doroftei@umfiasi.ro

**Russia**

Dr Vladislav Korsak, International Center for Reproductive Medicine, General Director, Liniya 11, Building 18B, Vasilievsky Island, 199034 St-Petersburg, Russia C.I.S. Tel: +7 812 328 2251; Fax: +7 812 327 19 50; Mobile: +7 921 9651977; E-mail: korsak@mcrm.ru

**Serbia**

Prof Snezana Vidakovic, Institute for Obstetrics and Gynecology, Clinical Centre ‘GAK’, Visegradska 26, 11000 Belgrade, Serbia. Tel: +381 63 24 23 80; E-mail: drvidakovicsnezana@gmail.com

**Slovenia**

Prof Borut Kovacic, Univerzitetni Klinicni Center Maribor, Department of Reproductive Medicine and Gynecological Endocrinology, Ljubljanska ulica 5, 2000 Maribor, Slovenia. Tel: +386 2 321 2160; Mobile: +386 31 211 711; E-mail: borut.kov@ukc-mb.si

**Spain**

Ms Irene Cuevas Sáiz, Hospital General Universitario de Valencia, Reproductive Medicine Unit. Av Tres Cruces, 2. 46014 Valencia, Spain. Tel: +34963 131 800; Mobile: +3467724565; E-mail: icuevassaiz@yahoo.es

Dr Fernando Prados Mondéjar, Hospital de Madrid-Montepríncipe, HM Fertility Center Monteprincipe, C/Montepríncipe 25, 28660 Boadilla del Monte, Spain. Tel: +34 917 089 931; Mobile: +34 646 737 237; E-mail: fernandojprados@gmail.com

**Sweden**

Prof Christina Bergh, Department of Obstetrics and Gynaecology, Sahlgrenska University Hospital, Bla Straket 6, 413 45 Göteborg, Sweden. Tel: +4631 3421000, +46736 889325; Fax: +4631 418717; Mobile: +46 736 889325; E-mail: Christina.bergh@vgregion.se

**Switzerland**

Ms Maya Weder, Administration FIVNAT, Postfach 754, 3076 Worb, Switzerland. Tel: +41 (0)31 819 76 02; E-mail: fivnat@fivnat-registry.ch

Dr med Marco Buttarelli, Centro Cantonale di Fertilità, Ospedale Regionale di Locarno ‘La Carità’, Via all’Ospedale 1, 6600 Locarno, Switzerland. Tel: +41 91 811 45 38; E-mail: fivnat@fivnat-registry.ch

**Turkey**

Prof Dr Mete Isikoglu, Gelecek Tup Bebek Merkezi, Caglayan Mh. Bulent Ecevit Bulvari 167, 07070 Antalya, Turkey. Tel: +90 554 2149493; Mobile: +90 242324526; E-mail: misikoglu@hotmail.com

Dr Basak Balaban, Amerikan Hastanesi—American Hospital, Assisted Reproduction Unit, Güzelbahçe Sok N° 20 Nisantesi, 34365 Istanbul, Turkey. Tel: +90 212 444 3 777; Fax: +90 212 311 2190; Mobile: +90 532 253 27 92; E-mail basakb@amerikanhastanesi.org

**UK**

Mr Richard Baranowski, Deputy Information Manager, Human Fertilization and Embryology Authority (HFEA), Finsbury Tower, 103-105 Bunhill Row, London EC1 Y 8HF, UK. Tel: +44 (0) 20 7539 3329; Fax: +44 (0) 20 7377 1871; E-mail: Richard.baranowski@hfea.gov.uk

**Ukraine**

Prof Dr Mykola Gryshchenko, IVF Clinic Implant Ltd, Academician V.I. Gryshchenko Clinic for Reproductive Medicine, 25 Karl Marx Str., 61000 Kharkiv, Ukraine. Tel: +380 57 124522; Fax: +380 57 705070703; Mobile: +380 57 705070703; E-mail: nggryshchenko@gmail.com
