# Supplementary material for: ART in Europe, 2018: results generated from European registries by ESHRE
Source: Hum Reprod Open. 2022 Jul 5;2022(3):hoac022. doi: 10.1093/hropen/hoac022 (PMC9252765; doi:10.1093/hropen/hoac022)
Supplement: hoac022_Supplementary_Data [file hoac022_supplementary_data.doc]

**Participating countries and Centres**

**Albania**

Tirana: Klinika Gliozheni

**Armenia**

Yerevan: Fertility Center; Vitromed; Shengavit; IVF Center of Institute of Perinatal Reproductive Medicine and Ob/Gyn; IVF Center of Institute of Maternal and child Health institute; IVF Center of Astkhik Medcial Center.

**Austria**

Baden: Babywunschklinik Glück GmbH

Bregenz: IVF-Zentren Prof. Zech

Dobl: Kinderwunschinstitut Schenk

Feldkirch: Kinderwunschzentrum Landeskrankenhaus Feldkirch

Graz: Institut für In-Vitro-Fertilisierung und Endokrinologie;  Institut für Hormonstörungen Wechselbeschwerden und Kinderwunsch; A. ö. Landeskrankenhaus Graz

Innsbruck: Landeskrankenhaus-Universitätskliniken Innsbruck; Private Kinderwunschklinik Dr. Josef Zech GmbH; WOMED Therapiezentrum Kinderwunsch GmbH

Klagenfurt: Sterignost Kinderwunschbehandlungs GmbH

Krumpendorf: Privatkrankenanstalt Parkvilla

Linz: Kepler Universitätsklinikum GmbH – Med Campus IV,

Oberpullendorf: A. ö. Krankenhaus Oberpullendorf

Spital an der Drau: Kinderwunschinstitut Dr. Kaimbacher

St. Pölten: Tiny Feet Kinderwunschklinik

Salzburg: IVF-Zentren Prof. Zech Salzburg GmbH; Landeskrankenhaus Salzburg – Universitätsklinikum der PMU; Babywunsch-Klinik Dr. Zajc GmbH

Tulln: Kinderwunsch im Zentrum

Vienna: Allgemeines Krankenhaus der Stadt Wien;  Goldenes Kreuz; GYNANDRON Dr. Freude; Wunschbaby-Zentrum, Institut für Kinderwunsch; Die KinderWunschKlinik; Fertilitätszentrum Döbling

Wiener Neustadt: Tiny Feet Kinderwunschklinik

Wels-Thalheim: Die KinderWunschKlinik Dr. Loimer GmbH; IVF- und Kinderwunschinstitut Prof. Dr. Tews GmbH & Co KG

**Belarus**

Minsk: Center of assisted reproduction "Embryo"; GU Republican Scientific Practical Center “Mother and Child”; EVA CLINIC IVF; City Clinical Maternity Hospital #2; Multi-field Medical Company “LODE”

Gomel: Public health institution «Gomel Regional Diagnostic Center of Medical Genetics with consultation "Marriage and Family"

Vitebsk: Family Health Center “Bina”

**Belgium**

Antwerpen: Centrum voor Reproductieve Geneeskunde, Algemeen Ziekenhuis Middelheim

Braine - L’alleud : Centre de Fécondation C.H. Interregional Edith Cavell (CHIREC),

Brugge: CRG – Brugge-Kortrijk, Algemeen Ziekenhuis Sint-Jan

Brussel: Centrum voor Reproductieve Geneeskunde, UZ Brussel; Clinique de Procréation Médicalement Assistée, Hôpital Universitaire Saint- Pierre - U.L.B.; Service de Gynécologie, Cliniques Universitaires Saint-Luc - U.C.L.; Centre de FIV de l’U.L.B. – Hôpital Erasme

Charleroi : Service Gyn/Obst GHdC, Clinique Notre Dame

Edegem: Centrum voor Reproductieve Geneeskunde, Universitair Ziekenhuis Antwerpen,

Genk: Genk Institute for Fertility Technology - GIFT, Ziekenhuis Oost-Limburg - St. Jan

Gent: Vrouwenkliniek- afdeling Reproductieve Geneeskunde, U.Z. – Gent; Centrum voor Fertiliteitstherapie, A.Z. Jan Palfijn

Leuven : Dienst Gynaecologie, Universitaire Ziekenhuizen KULeuven Gasthuisberg; Unit Reproductieve Geneeskunde, Regionaal Ziekenhuis Heilig Hart

Libramont: Centre d’Infertilité, Centre Hospitalier de l'Ardenne

Liege : CPMA-ULiège, Centre Hospitalier Régional de Liège

Namur : Service PMA, Centre Hospitalier Régional de Namur

Rocourt : Centre Liégeois pour l’étude et le traitement de la stérilité, Clinique Saint Vincent

**Bosnia and Herzegovina**

Banja Luka . Medical center « Medico-S », member of Pronatal group, Republic of Srpka, BiH

Tuzla: BH IVF Centre « Dr Balic »

**Bulgaria**

Blagoevgrad: Medical Center “Puls”

Burgas: UMBAL “Burgas”
Pleven: Medical Center “KIRM”; Medical Center “Repromed”

Plovdiv: Medical Center “Bora”; USBALAG “Selena”; SMCG “New Life”

Sofia: SAGBAL “Dr Shterev”; MBAL Vita; Medical Center “Afrodita” ; IVF Clinic “Todorovi Brothers” ; IVF Clinic Medical Center “Dimitrov” ; Medical Center “Nadejda Reproductiv Sofia”; SBALGAR “Malinov”; Medical Center “Reprobiomed”; PSAGBAL “St Sofia”; SBALAGRM “Sofia”; University Hospital “Lozenets”; SBALAG “Maichin Dom”; SBAL “St. Lazar”; Medical Center “Adela Fertility”; MBAL for women’s health “Nadejda”; ASMP-MC “Sofia – 2000”; Acibadem city clinic Tokuda, Clinic Medical Center “Vyara”; Medical Center “Technobios”; II SAGBAL „Sheinovo“; Medical Center „Dr. Lilia Dimitrova“

Ruse: Medical Center “Dr. Kunev”

Shumen: ASMP-MC “St. Ivan Rilski”

Stara Zagora: Medical Center “In vitro Trakia”

Tutrakan: Medical Center “St. Ivan Rilski”

Varna: AMCSMP “Maichin Dom”; MCAR “Varna”; MCRM “Radost”; MCRM “Nova radost”; ASMPMC “Olimed”; AMCSMP “Dr. Mario Davidkov”

**Czech Republic**

Brno: Gyn. - por. klinika FN Brno-CAR01; MUDr. Aleš Bourek, PhD.; Reprofit International S.r.o.; Reprogenesis; a.s. Repromeda, s.r.o.; Sanatorium Helios, s.r.o.; Unica, s.r.o.

Ceské Budejovice: pronatal repro, s.r.o.; sanatorium art, s. R.o.

Hradec kralove: sanus, s.r.o.

Jihlava: sanus jihlava

Karlovy vary: institut reprodukční medicíny a genetiky, s.r.o.; pronatal spa, s.r.o.

Kolin: pronatal genus

Kostelec nad orlicí: arleta ivf, s.r.o.

Liberec: gennet liberec

Olomouc: fakultni nemocnice olomouc, car, por.-gyn. Klinika; fertimed, s.r.o.; ivf clinic olomouc

Ostrava: eurofertil cz,a.s.; gyncentrum ostrava, s.r.o., ivf science ostrava a.s.; repromeda, s.r.o.

Pardubice: sanus pardubice

Plzen: fn plzeň - institut reprodukční medicíny; ivf - zentren prof. Zech - pilsen s.r.o.; natalart, s.r.o.

Praha: europe ivf international ; fakultní nemocnice v motole – car- gyn.- por. Klinika 2. Lf uk; ferticare se; fertilityport prague s.r.o.; gennet, s.r.o.; gennet,s.r.o.pobočka archa; gest, s.r.o.; gynem s.r.o.; iscare i.v.f., a.s.; ivf cube,s.r.o.; mmi prague s.r.o.; prague fertility centre; pronatal plus, s.r.o.; pronatal, s.r.o.; ústav pro péči o matku a dítě; všeobecná fakultní nemocnice v praze, car, gyn.- por. Klinika 1. Lf uk

Teplice: pronatal nord, s.r.o; stellart s.r.o.

Zlín: ivf czech republic, s.r.o.

**Denmark**

Aalborg: Fertilitetsklinikken Aalborg Universitets Hospital;

Aarhus: Aagaard Fertilitetsklinik; Privathospitalet Ciconia; Maigaards Fertilitetsklinik

Copenhagen: Dansk Fertilitetsklinik; Copenhagen Fertility Center; Fertilitetsklinikken Herlev Hospital; Fertilitetsklinikken Hvidovre Hospital; Fertilitetsklinikken Rigshospitalet; Fertilitetsklinikken Trianglen; Junoklinikken; VitaNova; Stork IVFklinik

Fredericia: IVF-SYD

Horsens: Fertilitetsklinikken Regionshospitalet Horsens

Holbæk: Fertilitetsklinikken Holbæk Sygehus

Odense: Fertilitetsklinikken Odense Universitetshospital; Odense IVF-Klinik

Skive: Fertilitetsklinikken Skive Sygehus

**Estonia**

Tallinn: East Tallinn Central Hospital Women’s Clinic; Nova Vita Clinic; West Tallinn Central Hospital Women's Center for Reproductive Medicine; Fertility Clinic Nordic

Tartu: Tartu University Hospital's Women's Clinic; Clinic Elite

**Finland**

Helsinki: Dextra Helsinki; Felicitas Mehiläinen Helsinki; Ovumia Fertinova, Helsinki University Central Hospital

Joensuu: Northern Carelia Central Hospital

Jyväskylä: Ovumia Fertinova Jyväskylä

Kuopio: inOva; Kuopio University Central Hospital

Lappeenranta: Felicitas Mehiläinen Lappeenranta

Oulu: Felicitas Mehiläinen Oulu; Oulu University Central Hospital

Tampere: Ovumia Fertinova Tampere; Tampere University Central Hospital

Turku: Aura; Felicitas Mehiläinen Turku; Turku University Central Hospital

**France**

Aix en Provence : CH du pays d’Aix

Amiens: Groupe Santé Victor Pauchet

Angers: C.H.U. D’ Angers

Ars Laquenexy: CHR de Mercy

Avignon: Polyclinique Urbain V

Bagnolet: Centre médicochirurgical Floréal

Bayonne: Capio Clinique Belharra

Beaumont : Clinique La Chataigneraie

Besancon: CHU Jean Minjoz; Polyclinique de Franche-Comté

Bondy : Hôpital Jean Verdier

Bordeaux : CHU Centre Aliénor d’Aquitaine

Brest : CHRU hôpital Morvan ; Clinique Pasteur Lanroze

Bron : Hôpital femme Mère Enfant

Bruges: Polyclinique Jean Villar

Caen: CHU de  Caen

Calais : CH de Calais

Chambray-Les-Tours: Pôle de Santé-Léonard de Vinci

Charleville-Mezieres: CHI Nord-Ardennes

Clamart : APHP Hôpital Antoine Béclère

Clermont-Ferrand : CHU Estaing

Contamine-sur-arve: CH ALPES LEMAN

Créteil: CHI de Creteil

Dijon : Complexe Hospitalier du Bocage

Dreux : CH Dreux hôpital Victor Jousselin

Ecully : Clinique du Val d'ouest Vendome

Epinal : Polyclinique la ligne bleue

Ermont : Capio clinique Claude Bernard

Fort-de-France : Clinique Saint-Paul

Guilherand-Granges: Clinique Pasteur

La Roche sur Yon: Clinique Saint-Charles

La Rochelle: Clinique du Mail

La Tronche: Hôpital couple enfant

Le Blanc Mesnil: Hopital Privé De La Seine Saint Denis

Le Chesnay: Centre Medico Chirurgical de Parly II

Le Mans: Clinique du Tertre Rouge

Le Port: Clinique Jeanne d'Arc

Lens : CH Lens

Les Abymes : CHU de Pointe à Pitre Abymes

Lille : Hôpital Jeanne de Flandres ; hôpital prive Le Bois

Limoges : Hôpital de la mère et de l’enfant

Lorient : CH Bretagne Sud

Lyon: Clinic Natecia

Marseille: APHM Hôpital de la Conception ; Clinique Bouchard ; Hôpital St Joseph

Meaux : CH de Meaux

Montivilliers : Groupe hospitalier du Havre

Montpellier : CHU Hôpital Arnaud de Villeneuve; Polyclinique Saint Roch

Mulhouse : Fondation du Diaconat

Nancy : Polyclinique Majorelle; CHRU Nancy maternité

Nantes : Hôpital femme enfant adolescent ; Clinique Brétéché Viaud; Clinique Jules Verne

Neuilly sur Seine: Hôpital Américain ; Centre Chirurgical Pierre Cherest ;

Nice: Clinique Saint Georges ; Hopital De L'archet

Nimes: CHU de Nimes  Hôpital Caremeau

Orleans: CHRO Hôpital de la source

Paris: Hopital Des Diaconesses; Hopital Pierre Rouques « Les Bluets » ; Hôpital Cochin-Hôtel Dieu-Broca;  Institut mutualiste Montsouris ; Clinique de La Muette ; APHP Hopital Bichat Claude Bernard; APHP Hôpital Tenon

Pau: Polyclinique de Navarre

Perigueux: Clinique Francheville

Perpignan: Clinique Saint Pierre

Poissy: C.H. Int. De Poissy/St Germain en Laye

Poitiers: CHU de Poitiers

Quint-Fonsegrives : Clinique Capio La Croix du Sud

Reims: Polyclinique de Courlancy; CHU de Reims Hopital Maison Blanche

Rennes: CHU de Rennes Hôpital Sud; Clinique mutualiste La Sagesse

Roanne: CH De Roanne

Rouen: CHU de Rouen Hôpital Charles Nicolle ; Clinique Mathilde Rouen

Saint-Cloud: CH des Quatre villes site St Cloud

Saint-Denis: CH General Delafontaine

Saint-Herblain: Polyclinique de L’Atlantique

Saint-Martin-Boulogne : CMCO Côte D’Opale

Saint-Martin- d’Heres: Clinique Belledonne

Saint Pierre : Groupe Hospitalier Sud Réunion

Saint-Priest-en-Jarez : CHU de Saint Etienne Hôpital Nord

Salouel: CHU Amiens Sud

Saran : Polyclinique es longues allées

Schiltigheim : SIHCUS/CMCO

Senlis: GHPSO de Senlis

Suresnes: Hôpital Foch

Toulon : Clinique Saint Michel

Toulouse : Hôpitaux Mère & Enfants

Tours : C.H.R.U. de Tours hôpital Bretonneau

Valenciennes-Saint-Saulve : Centre d’AMP de la polyclinique du Parc

Villeurbanne: Médipole Lyon-Villeurbanne

Vitry Sur Seine: Hôpital Privé de Vitry Site Noriets

**Germany**

Aachen: Kinderwunschzentrum Aachen

Aachen: Klinik für Gynäkologische Endokrinologie und Reproduktionsmedizin; Uniklinik - RWTH Aachen

Aalen: Kinderwunschzentrum Aalen

Amberg: Kinderwunschzentrum Amberg, Am Klinikum St Marien Amberg

Augsburg: Kinderwunschzentrum Augsburg, GMP

Bad Münder: Deutsche Klinik Bad Münder – Hannover, MVZ wagnerstibbe für Gynäkologie, Reproduktionsmedizin, Zytologie, Pathologie und Innere Medizin

Bad Schwartau: Kinderwunsch Holstein

Bayreuth: MVZ Fertility Center Bayreuth GmbH

Berlin: Wunschkinder Berlin; Ceres – Kinderwunschzentrum Dr. Hannen und Dr. Stoll; MVZ Fertility Center Berlin, Auf dem Gelände der DRK Kliniken Westend; Kinderwunschärzte Berlin GbR, Zentrum für Kinderwunschbehandlung und Fertilitätsprotektion; Kinderwunschzentrum am Innsbrucker Platz Berlin; Kinderwunschzentrum am Potsdamer Platz; Kinderwunschzentrum an der Gedächtniskirche; Praxis für Kinderwunschtherapie Helle-Mitte; Praxis für Fertilität, Gynäkologische Endokrinologie und Reproduktionsmedizin; MVZ Wunschkinder Berlin GmbH; MVZ TFP Berlin GmbH

Bielefeld: FROG – Kinderwunschinstitut und Frauenarztpraxis in der Praxisklinik Prof. Volz; Bielefeld Fertility-Center, Zentrum für Reproduktionsmedizin und Gynäkologische Endokrinologie

Bonn-Bad Godesberg: Praxisklinik für Gynäkologische Endokrinologie und Reproduktionsmedizin

Bonn: Kinderwunschzentrum Bonner Bogen; MVZ für Frauenheilkunde und IvF-Medizin Bonn GbR; Universitätsklinikum Bonn; Gynäkologische Endokrinologie und Reproduktionsmedizin

Bremen: Bremer Zentrum für Fortpflanzungsmedizin (BZF); Kinderwunsch Bremen

Bühl: Klinikum Mittelbaden; Zentrum für Minimal Invasive Gynäkologie, Endometriose und Reproduktionsmedizin

Chemnitz: Kinderwunschzentrum Praxisklinik City Leipzig; Standort Chemnitz

Darmstadt: Kinderwunschzentrum Darmstadt

Deggendorf: Kinderwunschzentrum Niederbayern

Dortmund: Überörtliche Berufsausübungsgemeinschaft Kinderwunsch Dortmund, Siegen, Dorsten, Wuppertal GbR

Dresden: Gynäkologische Endokrinologie und Reproduktionsmedizin der Universitätsfrauenklinik Dresden; Universitäres Kinderwunschzentrum; Kinderwunschzentrum Dresden; Praxisklinik Dr. med. H.-J. Held

Düsseldorf: KinderwunschKö; MVZ VivaNeo Kinderwunschzentrum Düsseldorf GmbH; UniKiD; Universitäres interdisziplinäres Kinderwunschzentrum Düsseldorf

Erlangen: GMP Dres. Hamori, Behrens, Hammel; Kinderwunschzentrum Erlangen; Universitäts-Fortpflanzungszentrum Franken (UFF)

Essen: novum - Zentrum für Reproduktionsmedizin; Überörtliche Gemeinschaftspraxis

Esslingen: IVF-Zentrum Esslingen

Frankfurt am Main: Gynäkologische Endokrinologie und Reproduktionsmedizin Re∙Pro∙Gyn; Universitätsklinikum Frankfurt am Main; Kinderwunsch & Hormonzentrum Frankfurt am Main; repromedicum Kinderwunschzentrum; Reproduktionsmedizin - Gyn. Endokrinologie

Freiburg: CERF Centrum für Gynäkologische Endokrinologie & Reproduktionsmedizin Freiburg; Universitätsklinikum Freiburg; Klinik für Frauenheilkunde, Endokrinologie und Reproduktionsmedizin

Garching b. München: Kinderwunschpraxis München Nord

Gelsenkirchen: Kinderwunschpraxis Gelsenkirchen; Wissenschaftspark Pav. 8, 1.OG

Göttingen: MVZ Kinderwunschzentrum Göttingen; Zentrum für Kinderwunsch und Reproduktionsmedizin; gyn-medicum Göttingen

Grevenbroich: green-ivf; Grevenbroicher Endokrinologie- und IVF-Zentrum

Großhansdorf: Universitäres Kinderwunschzentrum Lübeck und Manhagen; Zentrum für Gynäkologische Endokrinologie und Reproduktionsmedizin am Universitätsklinikum Schleswig-Holstein, Universitäre Kinderwunschzentren GmbH, Standort Manhagen

Hagen: Freyja IVF Hagen; Kinderwunsch & Hormonzentrum

Halle (Saale): Universitätsklinikum Halle (Saale); Zentrum für Reproduktionsmedizin und Andrologie

Hamburg: amedes experts; Facharzt-Zentrum für Kinderwunsch, Pränatale Medizin, Endokrinologie und Osteologie Hamburg; Kinderwunsch Praxisklinik Fleetinsel Hamburg; Kinderwunsch Valentinshof; Kinderwunschzentrum Altonaer Straße (MVZ) im Gynäkologicum Hamburg (GbR); KinderwunschZentrum HAFENCITY Hamburg; Medizinisches Versorgungszentrum; Fertility Center Hamburg GmbH

Hannover: Medizinische Hochschule Hannover; Klinik für Frauenheilkunde und Geburtshilfe, Abteilung Reproduktionsmedizin und gynäkologische Endokrinologie; Team Kinderwunsch Hannover

Heidelberg: Kinderwunschzentrum Heidelberg; Universitäts-Frauenklinik Heidelberg; Abt. Gynäkologische Endokrinologie und Fertilitätsstörungen

Hildesheim: Zentrum für Reproduktionsmedizin & Humangenetik; MVZ

Homburg: Klinik für Frauenheilkunde, Geburtshilfe und Reproduktionsmedizin; Universitätsklinikum des Saarlandes

Jena: Klinik und Poliklinik für Frauenheilkunde und Fortpflanzungsmedizin; Kinderwunsch und Hormonzentrum; Zentrum für Reproduktionsmedizin Jena & Erfurt; Gemeinschaftspraxis Dres. Friztsche

Karlsruhe: Kinderwunschzentrum Karlsruhe

Kassel: MVZ für Reproduktionsmedizin am Klinikum Kassel

Kempten: KinderWunschKempten; Zentrum für Reproduktionsmedizin am Klinikum Kempten, Klinikverbund Kempten-Oberallgäu

Kiel: fertilitycenterkiel; Kinderwunsch Kiel; Universitäres Kinderwunschzentrum Kiel

Köln: Klinik und Poliklinik für Frauenheilkunde und Geburtshilfe der Universität zu Köln, Gynäkologische Endokrinologie und Reproduktionsmedizin; MVZ amedes; Praxis und Klinik Schönhauser Straße PKS; MVZ PAN Institut für Endokrinologie und Reproduktionsmedizin GmbH; Zentrum für Kinderwunschbehandlung Köln

Langenhagen: Kinderwunschzentrum Langenhagen & Wolfsburg MVZ

Leer (Ostfriesland): Kinderwunschzentrum Ostfriesland

Leipzig: Kinderwunschzentrum Leipzig-Chemnitz; Standort Leipzig; Kinderwunschzentrum Praxisklinik City Leipzig; Standort Leipzig

Lübeck: Universitäres Kinderwunschzentrum Lübeck und Manhagen; Zentrum für Gynäkologische Endokrinologie und Reproduktionsmedizin am Universitätsklinikum Schleswig-Holstein, Universitäre Kinderwunschzentren GmbH, Standort Lübeck

Ludwigsburg: Kinderwunschzentrum Ludwigsburg

Ludwigshafen: Kinderwunschzentrum Ludwigshafen

Magdeburg: Kinderwunschzentrum Magdeburg; Otto-von-Guericke-Universität Magdeburg; Klinik für Reproduktionsmedizin

Mainz: Kinderwunsch Zentrum Mainz; Kinderwunschzentrum der Universitätsmedizin Mainz

Mannheim: Kinderwunschzentrum der Universitätsmedizin Mannheim

Marburg: Reproduktionsmedizinisches Kompetenzzentrum Marburg und Gießen

Minden: Zentrum für Kinderwunschbehandlung und pränatale Medizin; GMP

Mönchengladbach: proKindwunsch; Kinderwunschzentrum Niederrhein

München: A.R.T. Bogenhausen MVZ; Hormon- und Kinderwunschzentrum; Klinik und Poliklinik für Frauenheilkunde und Geburtshilfe der LMU München; Kinderwunsch Centrum München; (MVZ); Kinderwunsch Zentrum an der Oper; kïz) kinderwunsch im zentrum; Praxis für gynäkologische Endokrinologie und Reproduktionsmedizin; Reproduktionsmedizin München - MVZ

Münster: Kinderwunschpraxis an der Promenade; GMP Mempel & Stratmann; MVZ Kinderwunsch und Hormonzentrum Münster GmbH; Kinderwunschzentrum Münster; UKM Kinderwunschzentrum; Universitätsklinikum Münster

Neckarsulm: Kinderwunschzentrum Neckarsulm; KinderwunschFrauenaerzte

Neuwied: Kinderwunschzentrum Mittelrhein

Nürnberg: Kinderwunsch und Frauen-Hormon Centrum Nürnberg

Offenbach: Kinderwunsch und Endometriosezentrum am Büsing Park

Oldenburg: Tagesklinik Oldenburg; Team Kinderwunsch Oldenburg

Osnabrück: Zentrum für Kinderwunschbehandlung Osnabrück

Pforzheim: Centrum für Kinderwunsch Pforzheim

Potsdam: Kinderwunschzentrum Potsdam

Prien am Chiemsee: Kinderwunsch Centrum Chiemsee

Recklinghausen: REProVita; Kinderwunschzentrum Recklinghausen

Regensburg: MVZ KITZ Regensburg GmbH; profertilita ; Fachklink für Fruchtbarkeitsmedizin

Remscheid: Bergisches Kinderwunschzentrum Remscheid

Rostock: Praxis für Fertilität; MVZ GmbH

Saarbrücken: IVF-SAAR Saarbrücken-Kaiserslautern

Singen: Kinderwunsch Bodensee

Stuttgart: Kinderwunsch-Zentrum Stuttgart; Kinderwunsch-Zentrum Stuttgart; Praxis Villa Haag

Trier: Kinderwunsch Praxisklinik Trier; Wissenschaftspark (WIP)

Tübingen: IVF-Zentrum der Universitäts-Frauenklinik Tübingen; KinderwunschPraxis Dres. Göhring

Ulm: Kinderwunsch-Zentrum Ulm; Praxisklinik Frauenstraße Ulm; MVZ für Kinderwunsch und Pränatalmedizin; Universitätsfrauenklinik Ulm; UniFee – Kinderwunsch / Fertility and Endocrinology

Viernheim: Viernheimer Institut für Fertilität

Wetzlar: Kinderwunschzentrum Mittelhessen

Wiedbaden: MVZ Vivaneo Kinderwunschzentrum Wiesbaden GmbH

Würzburg : MainKid; Kinderwunschzentrum am Theater; Universitätsklinikum Würzburg; Frauenklinik und Poliklinik, Zentrum für gynäkologische Endokrinologie und Reproduktionsmedizin (ZERM); Zentrum für Reproduktionsmedizin, Medizinische Genetik, Pränataldiagnostik

**Greece**

Attiki - Athens: University Hospital Aretaieion, IVF unit - Embryogenesis IVF unit - Eugonia IVF unit - Akeso IVF UNIT  - IVF Athens Center - Gennima IVF unit - Neogenesis IVF unit - Embryoart IVF unit - Genesis Athens, IVF unit - Embryoland IVF unit - Iakentro Athens - Iaso Athens, IVF unit - Biodimiourgia, IVF Unit - Institouto Gonimothtas - Mitosis IVF unit - Kapetanakis IVF Center - Kentro Biogenetikhs - Medimall IVF Unit - Monada Anaparagogikis Iatrikis IVF Unit - Mitrotis IVF UNIT  - Assisted Reproduction Unit -  emBIO IVF unit -  Maternity Health Unit - General Hospital Athens ALEXANDRA - University Hospital Attikon, IVF Unit - Mitera Hospital, IVF Unit -  Rea Maternity, IVF unit - Serum IVF Unit - University hospital Elena Benizelou - Titani Ziogas Vasileios IVF Unit

Crete: Mediterranean Fertility Institute  IVF UNIT Crete - Crete Fertility Centre ivf unit

Ioannina: University Hospital Ioanninon, IVF unit

Patra: Genesis Fertility centre

Thessaloniki: University Hospital Papageorgiou IVF unit -  Thessaloniki IVF center  - Iakentro Thessaloniki -  Assisting Nature IVF Unit - Biogenesis IVF Unit -  Embryoclinic, IVF unit - Embryolab, IVF unit -  Newlife SA, IVF unit - Genesis Fertility IVF unit  - Fertility Clinic- Interbalkan medical centre

Thessaly: University Hospital Thessalias, IVF Unit - Iaso Thessalias, IVF unit

Thrace: Embryokosmogenesis IVF Unit

**Hungary**

Budapest: Division of Assisted Reproduction, Dept. of OB/GYN, Semmelweis University, Faculty of Medicine; Buda Infertility Center, St. John’s Hospital; Sterility Devai Institute; Forgacs Institute; Róbert Károly Infertility Center; Kaáli Institute; Versys Clinics Human Reproduction Institute; Reprosys Reproduction Center

Debrecen: Assisted Reproduction Center, University of Debrecen

Gyõr: Kaáli Institute

Kaposvár: Kaposi Mór Teaching Hospital

Pécs: Departement of OB/GYN, University of Pécs

Szeged: Kaáli Institute, Pannon Reproduction Institute

Tapolca: Kaáli Institute

**Iceland**

Reykjavik: Livio Reykjavik

**Ireland**

Dublin: Beacon Care Fertility; Merrion Fertility Clinic

**Italy**

Acerra: Villa dei Fiori s.r.l.

Ancona: Centro PMA - Presidio Ospedaliero “G. Salesi”.

Aosta: Centro PMA Valle d’Aosta.

Appiano Gentile: Le Bettulle Casa di Cura s.r.l. - Unità di Procreazione Medico Assistita

Arco: Centro Provinciale per la Procreazione Medicalmente Assistita - Ospedale Alto Garda e Ledro.

Asti: Centro di Medicina della Riproduzione - SOC Ostetricia e Ginecologia dell'Ospedale Cardinal Massaia.

Avellino: Fisiopatologia della Riproduzione e Sterilità di Coppia - A.O.R.N. San Giuseppe Moscati di Avellino.

Bari: Centro Medico "San Luca"; Centro PMA - Casa di Cura Santa Maria.

Barletta: Pro Andros s.r.l.

Battipaglia: Embryos S.r.l.

Benevento: Centro di Fisiopatologia della Riproduzione Umana - AORN "G. Rummo" di Benevento.

Bergamo: Centro di Fisiopatologia della Riproduzione - A.O. Papa Giovanni XXIII.

Bisceglie: MOMO' FertiLIFE.

Bologna: SISMER - Soc. Italiana Studio Med. Ripr.; Str. Sempl. Dipt. di Infertilità e PMA - Centro di riferimento regionale per la preservazione della fertilità nei pazienti oncologici - Dipt. Attività Integrata del Bambino, della Donna e delle Malattie Urologiche - AOU Policlinico S. Orsola Malpighi; Tecnobios Procreazione s.r.l.; Poliambulatorio Privato Day Surgery GynePro Medical.

Bolzano: Donna Salus.

Bra: Centro PMA - Casa di Cura "Città di Bra".

Brescia: Centro di Fecondazione Medicalmente Assistita - U.O. Ostetricia e Ginecologia - Casa di Cura "Istituto Clinico Città di Brescia"

Brindisi: Progenia S.r.l.; Casa di Cura SALUS s.r.l.

Brunico: Centro di medicina della riproduzione umana e crioconservazione gameti - Ospedale di Brunico.

Cagliari: Centro per la Diagnosi e Cura della sterilità di Coppia - Università degli Studi di Cagliari - Ospedale S. Giovanni di Dio di Cagliari; Servizio Ostetricia e Ginecologia - Diagnosi Prenatale e Preimpianto - Opedale Regionale Microcitemico di Cagliari.

Cantù: Centro di Fisiopatologia della Riproduzione del P.O. di Cantù - ASST LARIANA.

Carmagnola: Policlinico San Luca s.r.l.

Caserta: Genesis Day Surgery e sterilità della Coppia s.r.l.; CARAN srl - Medicina e Biologia della Riproduzione.

Cassino: Centro PMA del Cassinate.

Catania: Azienda Ospedaliera Universitaria Vittorio Emanuele - Presidio Ospedaliero Santo Bambino - Centro di Fisiopatologia della Riproduzione Umana; C.R.A. Centro di Riproduzione Assistita s.r.l.; Centro di Medicina della Riproduzione; Centro di Ginecologia e Medicina della Riproduzione GmR di Giuseppe Iraci Sareri; Casa di Cura Falcidia srl; A.O. "Cannizzaro" - Centro di PMA; Arnas Garibaldi - Centro di PMA P.O. Garibaldi di Nesima.

Cattolica: U.O. Fisiopatologia della riproduzione umana - Ospedale Cervesi di Cattolica - Azienda USL della Romagna; Extra Omnes - Medicina e Salute Riproduttiva.

Cava de’ Tirreni: Artemisia H. S.r.l. - Casa di Cura R. Ruggiero;

Chianciano Terme: Chianciano Salute - Centro di Chirurgia Ambulatoriale.

Chieti: Casa di Cura Spatocco - Centro I.E.M.A. - Istituto Europeo Medicina della Riproduzione Abruzzese.

Città Sant’Angelo: Casa di Cura Villa Serena del Dott. L. Petruzzi s.r.l.

Cittadella: Centro di Fecondazione Medicalmente Assistita di Cittadella - U.O.A. Ostetricia e Ginecologia - Dipartimento Materno Infantile - P.O. di Cittadella.

Conegliano: Centro Regionale Specializzato di Fisiopatologia della Riproduzione - Ospedale Civile di Conegliano.

Conversano: U.O. Fisiopatologia della Riproduzione Umana e P.M.A.

Cortona: Centro Procreazione Medicalmente Assistita - Ospedale Valdichiana Santa Margherita.

Erice: Casa di Cura Sant'Anna Centro di PMA.

Fermo: Casa di Cura Palmatea - Centro satellite SISMER.

Firenze: Florence - Centro di Chirurgia Ambulatoriale ed Infertilità; SOD di Procreazione Medicalmente Assistita - Università degli Studi di Firenze - A.O. Careggi; Centro di Procreazione Assistita "Demetra"; Futura Diagnostica Medica - Procreazione Medicalmente Assistita s.r.l.

Forlì: Centro di Procreazione Medico Assistita Villa Serena.

Fossano: S.S. Fisiopatologia della Riproduzione Umana.

Frosinone: Centro Medico Life Srl.

Galliate: Centro Procreazione Assistita c/o Sede di Galliate.

Genova: Medicina della Riproduzione - Ospedale Evangelico Internazionale; UOS Fisiopatologia della Riproduzione Umana IRCCS Policlinico Ospedale San Martino Genova.

Gioia Tauro: Gatjc - Gioia Tauro

Giugliano In Campania: Clinic Center HERA – Centro HERA srl.

Gragnano: Studio A.G.O.I. del Dott. A. M. Irollo

Grosseto: Demetra - Centro Grossetano per la Cura dell'Infertilita'

Lamezia Terme: C.I.S. Medicina Della Riproduzione

L'Aquila: U.O.C. Ostetricia e Ginecologia D.U. (con Centro Fivet) - del P.O. dell'Aquila.

Latina: Ospedale Santa Maria Goretti - Dipartimento Materno lnfantile - UOC Ostetricla e Ginecologla DEA ll - UOS Fisiopatologia della Riproduzione (PMA).

Lecce: Casa di Cura “Prof Petrucciani” – Centro di Procreazione Medicalmente Assistita

Livorno: CRPO - Medicina della Riproduzione.

Lugo: Servizio di Fisiopatologia della Riproduzione Umana - AUSL della Romagna P.O. di Lugo.

Maddaloni: IATREION s.r.l.- Medicina Polispecialistica

Manerbio: Dipartimento di PMA - U.O. Ostetricia e Ginecologia - A.O. di Desenzano del Garda - P.O. di Manerbio.

Mantova: Centro di Medicina della Riproduzione ed Endocrinologia - U.O. Ostetricia e Ginecologia - A.O. “Carlo Poma”.

Marcianise: UOSD di Fisiopatologia della Riproduzione-P.O. di Marcianise- ASL Caserta

Marostica: Genera Veneto Srl.

**Kazakhstan**

Actobe: IVF Center " FAMILY DOCTOR AND CO",

Almaty: International Clinical Center for Reproductology “Persona”; Institute of Reproductive Medicine; ECOMED clinic; Nuray Clinic

Nur-Sultan: “Astana ECOLIFE”; Genom Clinic

Taraz: “Medical Center of Marriage and Family”

Shymkent: “IVF clinic of Dr.Tararaka”

Karaganda: LLP “Akzhan”

**Latvia**

Riga: Your Doctors clinic, RMC “Embrions”, My Clinic Riga

**Lithuania**

Vilnius: Vilnius University Hospital Santaros Klinikos Santaros Fertility Center, public clinic; Northway Medical Center, private clinic; **Grazinos Bogdanskienes Fertility Center, private clinic**

Klaipėda: „Jolsana“ Medical Center, private clinic.

**Vilnius/Kaunas/Klaipėda: Vaisingumo Klinika, private clinic.**

**Luxembourg**

Luxembourg: Centre Hospitalier de Luxembour, Centre de Stérilité et de Reproduction

**Malta**

Msida: Mater Dei Hospital ART clinic, public governmental centre for ART

Sliema: St James Hospital, private ART clinic

**Moldova**

Chisinau: Repromed clinic; TerraMed medical center; Medpark hospital

**Montenegro**

Budva: Human Reproduction Centre, Budva, private centre

Cetinje: Human Reproduction Department, Hospital Danilo I Cetinje;

Podgorica: LIFE, private IVF clinic;

Podgorica: ARS MEDICA, private IVF clinic

**North Macedonia**

Bitola: Private Hospital Plodnost

Skopje: First Private General Hospital - Centre for Assisted Reproduction and IVF; Acibadem Sistina Hospital - Centre for Assisted Reproduction (IVF); Newborn Clinic-Fertility Clinic

Stip: Private Health Centre D-r Organdziski

**Norway**

Bergen: Haukeland Universitetssykehus; Klinikk Hausken

Haugesund: Haugesund Sykehus HF; Klinikk Hausken

Oslo: Livio IVF klinikken Oslo; Oslo Universitetssykehus; Fertilitetssenteret; Klinikk Hausken; Medicus

Porsgrunn: Sykehuset Telemark HF

Stavanger: Klinikk Hausken ; Medicus

Tromsø: Universtitetssykehus Nord Norge

Trondheim: Spiren Fertilitetsklinikk; St Olavs Hospital ; Medicus

**Poland**

Białystok: BOCIAN Klinika Leczenia Niepłodności, Ginekologii i Położnictwa; Klinika Rozrodczości i Endokrynologii Ginekologicznej, Uniwersytecki Szpital Kliniczny; KRIOBANK Centrum Leczenia Niepłodności, Ginekologia i Położnictwo

Bydgoszcz: GENESIS NZOZ Centrum Medyczne

Bytom: ANTRUM Centrum Medyczne, Laboratorium DEMETER Stanisław Horák

Gdańsk: INVICTA Kliniki Leczenia Niepłodności

Gdynia: GAMETA Centrum Zdrowia; INVIMED Europejskie Centrum Macierzyństwa

Katowice: ANGELIUS PROVITA Centrum Medyczne; BOCIAN Klinika Leczenia Niepłodności, Ginekologii i Położnictwa; GYNCENTRUM Klinika Leczenia Niepłodności i Diagnostyki Prenatalnej; INVIMED Europejskie Centrum Macierzyństwa; ZIĘBA Clinic NZOZ

Kielce: GAMETA Centrum Zdrowia

Kraków: ARTVIMED Centrum Medycyny Rozrodu; MACIERZYŃSTWO Centrum Medyczne;

PARENS Centrum Leczenia Niepłodności

Łódź: GRAVITA Diagnostyka i Leczenie Niepłodności; SALVE-MEDICA

Lublin: AB OVO NZOZ Centrum Zdrowia Rodziny

Motycz: OVUM Specjalistyczne Centrum Medyczne

Mysłowice: NOVOMEDICA Centrum Leczenia Niepłodności

Niemcz: ZDRÓWKO Klinika

Olsztyn: ARTEMIDA Centrum Ginekologii, Endokrynologii i Medycyny Rozrodu

Opole: PARENS Centrum Leczenia Niepłodności

Płock: GRAVIDA

Poznań: BOCIAN Klinika Leczenia Niepłodności, Ginekologii i Położnictwa; INVIMED Europejskie Centrum Macierzyństwa; Klinika Niepłodności i Endokrynologii Rozrodu Uniwersytet Medyczny im. Karola Marcinkowskiego w Poznaniu; MEDART Ośrodek Diagnostyki i Leczenia Niepłodności

Rzeszów: PARENS

Rzgów: GAMETA Szpital

Szczecin: VITROLIVE Centrum Ginekologii i Leczenia Niepłodności

Warszawa: BOCIAN Klinika Leczenia Niepłodności, Ginekologii i Położnictwa; FERTIMEDICA Centrum Płodności; FERTINA Centrum Medyczne; INVICTA Kliniki Leczenia Niepłodności;

INVIMED Europejskie Centrum Macierzyństwa; nOvum Przychodnia Lekarska; Uniwersyteckie Centrum Zdrowia Kobiety i Noworodka WUM, I Klinika Położnictwa i Ginekologii arszawskiego Uniwersytetu Medycznego; GAMETA Centrum Zdrowia; SALVE-MEDICA

Wrocław: INVICTA Kliniki Leczenia Niepłodności; INVIMED Europejskie Centrum Macierzyństwa; POLMEDIS POLAK Klinika Leczenia Niepłodności

**Portugal**

Guimarães: Hospital da Senhora da Oliveira – Guimarães

Vila Nova de Gaia: Centro Hospitalar de Vila Nova de Gaia / Espinho, EPE - Unidade de Medicina da Reprodução Dra. Ingeborg Chaves

Vila Real: Centro Hospitalar de Trás-os-Montes e Alto Douro, EPE;

Porto: Centro Hospitalar do Porto, EPE; Centro Hospitalar de São João, EPE; Centro de Genética da Reprodução Prof. Alberto Barros; CETI (Centro de Estudos e Tratamento da Infertilidade); CEIE (Centro de Estudos de Infertilidade e Esterilidade)

Espinho: COGE (Clínica Obstétricia e Ginecológica de Espinho)

Braga: Ferticare (Centro de Medicina da Reprodução)

Coimbra: Centro Hospitalar Universitário de Coimbra, EPE; CLINIMER (Clínica de Medicina da Reprodução); FERTICENTRO (Centro de Estudos de Fertilidade)

Covilhã: Centro Hospitalar Cova da Beira, EPE;

Lisbon: Centro Hospitalar Lisboa Norte, EPE - Hospital de Santa Maria; Centro Hospitalar Lisboa Central, EPE - Maternidade Dr. Alfredo da Costa; AVA CLINIC; British Hospital XXI; CEMEARE; Hospital dos Lusíadas; Instituto Extremeño de Reproducción Asistida, Sucursal em Portugal (IERA); IVI Lisboa; MALO CLINIC - GINEMED Lisboa

Almada: Hospital Garcia de Orta, EPE

Faro: FERTIMED (Centro Médico de Reprodução Humana)

Ponta Delgada: MEKA CENTER (CLÍNICA DA MULHER)

Funchal: Hospital DR. Nélio Mendonça, Sesaram, EPE; FERTIMADEIRA (Centro de Estudos de Fertilidade e de Criopreservação da Madeira)

**Romania**

Bucuresti: MEDLIFE S.A.; LIFE LINE MEDICAL CENTER(Columna Medical Center); Clinica Medicala Gynera; PROMED SYSTEM( Wellborn Militari /Baneasa); Spitalul Clinic de Obstetrică și Ginecologie "Prof Panait Sarbu

Iasi: NEW LIFE – BM S.R.L.; Fertigyn SRL;

Sibiu: Imes-Optogyn, Polisano

Constanta: Euromaterna

Timisoara : IVF Clinic SL

**Russia**

Arkhangelsk: LLC "IVF Center"

Astrakhan: Region Center of Family Health Care and Reproduction

Barnaul: Siberian institute of human reproduction and genetics; Regional clinical hospital; Clinic "Mother and Child Barnaul"

Belgorod: Regional clinical hospital of St. Joasaph, Department of ART

Blagoveshchensk: LLC Clinic “AmurMed”

Bryansk: Regional Center of Family Health Care and Reproduction; Bryansk Interdistrict Hospital

Cheboksary: State-financed organization Presidential Perinatal Center of the Ministry of Health and Social Development of the Chuvash Republic

Chelabinsk: Municipal Autonomous health care institution "ART Center"; Regional Perinatal Center; LLC Medical Center "Lotos"; Center of Family Medicine; LLC "DNA clinic"; Clinic "Source"

Chita: Trans-Baikal regional perinatal center

Ekaterinburg: Ural Research Institute of Maternity and Child Care; Center of Family Medicine; Multifunctional Center "Harmony"; Clinical and diagnostic center "Maternal and child health protection", LLC Clinical Institute of Reproductive Medicine

Irkutsk: Regional perinatal center, Department of ART; Clinic "Mother and Child Irkutsk"

Ivanovo: Clinic of Modern Medicine; Research Institute of Maternity and Childhood n.a. V.N. Gorodkov

Izhevsk: Center for reproductive health; Clinic of Nuriev – Izhevsk; First Republican Clinical Hospital, Center of IVF and Reproduction

Kaliningrad: LLC "Center-Doctor"

Kaluga: Dr. Fomin's Clinic

Kazan: Clinic "Scandinavia AVA-Kazan"; LLC "Nuriev's clinic"; Kazan branch of LLC "AVA-PETER"; Clinic of Family Medicine

Kemerovo: LLC "Center for Family Health and Reproduction "Krasnaya Gorka"

Kirov: Regional Clinical Perinatal Center; LLC "Nuriev's clinic - Kirov"

Kislovodsk: Clinic «Elorma»

Khabarovsk: Perinatal Center n.a. Prof. G.S.Postol

Khanty-Mansiysk: District Clinical Hospital

Krasnodar: "OXY-Center” Ltd; Clinic of Human Reproduction “Embryo”; Clinic "First word"; Regional Center of Family Health Care and Reproduction; Base Obstetrics and Gynecology Clinic of Kuban State Medical University; LLC "Clinic Ekaterininskaya"; Children's City Clinical Hospital

Krasnoyarsk: Clinic "Mother and Child Krasnoyarsk"; Medical Center Gynecological Endocrinology and Reproduction «Three hearts»

Kurgan: JSC "Family Medicine Center"

Kursk: Regional perinatal center

Magnitogorsk: JSC "Family Medicine Center"; LLC " DNA CLINIC"

Makhachkala: Dagestan Republican Center of Family Health Care and Reproduction

Moscow: Moscow Regional Perinatal Center, ART department; JSC "European Medical Center", Clinic of Reproduction and prenatal Medicine; City Clinical Hospital n.a. V.V. Veresaev ", ART Department; Moscow Regional Research Institute of Obstetrics and Gynecology; Clinic "Moskvorechye"; Perinatal Medical Center "Mother and Child"; Medical Center for Infertility treatment" Embryo"; University Clinic "I am healthy!"; Family Planning and Reproduction Clinic "Intime”; VitroKlinik; GMS ECO; Institute of Reproductive Medicine REMEDI; Clinic "Mother and Child Lefortovo"; Clinic "Mother and Child Khodynka field"; Clinic of assisted reproductive technologies "Test tube baby"; Clinic "Mother and Child Savelovskaya"; LLC "Clinic of Professor V.M. Zdanovsky"; Center for Reproduction and Genetics “Nova Clinic”; Medical Center “ART-IVF”; Clinic "New life"; Clinic K+31 "Petrovskie Vorota"; NGC Clinic; Clinic MAMA; LLC "Prior Clinic"; Clinic "Biooptima"; Clinic "Mother and Child Kuntsevo"; Lapino Clinical Hospital; Reproduction Center "Lifeline"; Center of Reproduction and Genetics "Fertimed"; Clinic "Altra Vita"; First Moscow State Medical University n.a. I.M. Sechenov; National Medical Research Center of Endocrinology; National Medical Research Center of Obstetrics, Gynecology and Perinatology n.a. V.I.Kulakov", Department of Assistive Technologies in Infertility Treatment n.a. B.V.Leonov; National Medical Research Center of Obstetrics, Gynecology and Perinatology n.a. V.I.Kulakov", 1st gynecological department; Central Clinical Hospital with Polyclinic" of the Office of the President of the Russian Federation; Central Clinical Hospital of the Russian Academy of Sciences; Central Clinical Hospital "RZD-Medicine"

Naberezhnye Chelny: Clinic of Nuriev – Chelny

Nizhny Novgorod: Clinic «AIST»; Federal Budget Health Care Institution "Volga district health center"; Clinic “Dad, mom and baby”

Novokuznetsk: Novokuznetsk City Clinical Hospital No. 1; Clinic "Media-Service"; Clinic "Mother & Child Novokuznetsk”

Novosibirsk: Medical Center "Avicenna"; Center for reproductive medicine “Mother and Child”; Professor Pasman's Clinic

Omsk: The fertility clinic "EmBio"; City clinical perinatal center; Omsk Center for Reproductive Medicine; Multidisciplinary Center of Modern Medicine "Euromed"

Orenburg: Regional Clinical Hospital No. 2; Medical Center for Cell Technologies “New Life”; Clinic “Medgard-Orenburg"; Clinic "MaxiMed"

Penza: LTd “INMED”

Perm: Reproductive Clinic “Philosophy of Life”; Clinic “Mother and Child Perm”

Rostov-Don: Center of Human Reproduction and IVF; Clinic «Genom-Don»

Ryazan: Clinic "Mother and Child Ryazan"

Samara: State-Financed Health Facility "Samara Regional Medical Center “Dynasty"; IDK Medical Company - Clinic "Mother and Child Samara"; Clinical Hospital "IDK"; Clinic for reproductive health “IVF”

Saransk: Mordovian Republican Clinical Perinatal Center; "Centre IVF "Gera", LLC

Saratov: Clinical Perinatal center of the Saratov region; Clinic of doctor Paramonov; Professor Churakov's Clinic; Center for Reproductive Technologies "SOVA IVF"

Simferopol: Clinic "Vera"; Genesis Clinic

Sochi: The Russian-American center for reproduction and human genetics; Medical Center “Embryo-Sochi”

St.Petersburg: International Center for Reproductive Medicine; City Mariinsky Hospital, ART department; Saint-Petersburg State Establishment of health protection «Maternity welfare clinic №44» of Pushkinskiy district; Reproduction and family planning center; Saint-Petersburg State Pediatric Medical University; Baltic Institute of Human Reproductology; “AVA-PETER” Ltd; "Aimed" Ltd; Federal Medical research centre n.a. V. A. Almazov; Euromed-clinic; «Genesis» Ltd; Clinical Hospital # 122 n.a. L.G.Sokolov; Clinic “EmbryLife”; Next Generation Clinic; Clinic "Mother and Child St.Petersburg"; The family planning center "Medica"; Military Medical Academy n.a. S.M. Kirov; Federal state budgetary scientific institution "Research Institute of obstetrics, gynecology and reproduction n.a. D.O.Ott”, Department ART; LLC “ICLINIC”

Stavropol: Stavropol Regional Clinical Advisory and Diagnostic Center

Surgut: Surgut Clinical Perinatal Center

Syktyvkar: Komi Republican Perinatal Center

Tolyatti: Consultative and diagnostic department Interdistrict perinatal center GBUZ CO "TGKB N 5"; Clinic "Mother and Child Tolyatti"

Tomsk: Regional Perinatal Center n.a. I.D. Yevtushenko, ART department

Tver: Dr. Fomin's Clinic; Сlinical perinatal center n.a. E.M. Bakunina; Clinic of reproductive medicine and genetics "Genetis"

Tula: Center of new medical technology; VitroClinic

Tumen: International Medical Center for Reproduction "Mercury"; Medical Center "Malysh"; Clinic "Mother and Child Tumen"

Ulan-Ude: DiaGroup Medical Center LLC

Ulyanovsk: Аlliance Сlinic LLC

Ufa: Republican Medical and Genetic Center; Medical Center “Family”; Clinic "Health of women and men"; Clinical hospital "Mother and Child. Ufa"

Vladikavkaz: Republic Center of Family Health Care and Reproduction

Vladivostok: LLC Women's Health Clinic "Santa Maria"; Regional Clinical Center for Specialized types of medical care

Vladimir: Clinic "Mother and Child Vladimir"

Volgograd: Volgograd State Medical University, Clinic №1; Clinic “Genom-Volga”; Clinic "Mother and Child Volgograd”

Voronezh: Voronezh Region Clinical Hospital №1; Clinic "Mother and Child Voronezh”

Yakutsk: Republic Hospital N1 – National Centre of Medicine, Department of ART

Yaroslavl: Regional Perinatal Center; Clinic "Mother and Child Yaroslavl"; Medical center "Origin"

**Serbia**

Belgrade: Klinika za ginekologiju i akušerstvo Klinički Centar Srbije; Specijalna bolnica za lečenje steriliteta "Intermedicus BIS", Specijalna ginekoloska bolnica SRETENOVIC, Beograd;

**Slovenia**

Ljubljana: Department of Human Reproduction, Division of Gynaecology, University Medical Centre Ljubljana,

Maribor: Department of Reproductive Medicine and Gynaecologic Endocrinology, University Medical Centre Maribor,

Postojna: Centre for Infertility Treatment Postojna

**Spain**

A Coruña: Equipo Ron – Hospital Quirón A Coruña; HM Belén; Clínica Segrelles

Albacete: Consultorio de Ginecología y Obstetricia; H. General De Albacete; Instituto Bernabéu ALBACETE

Alcalá De Henares: Dr. Goya Analisis S.L.; H. Principe De Asturias; Unimequi

Alcázar de San Juan: Ginequalitas SLP

Alcorcón: ReproFiv

Algeciras: Clínica Medrano

Alicante: Ferrobelab, S.L.; H. General De Alicante; Instituto Bernabéu; IVF-SPAIN (Alicante); IVI Alicante; Unidad de Reproducción Clínica Vistahermosa; Accuna.

Almería: C.H. Torrecárdenas; IVI Almeria; Unidad de Reproducción - Hospital Mediterráneo

Almoradí: Clínica Ufeal

Aravaca: URH Garcia Del Real

Badajoz: CERHA; Instituto Extremeño de Reproducción Asistida

Baracaldo: Hospital Universitario Cruces

Barcelona: C.I.R.H; Centro de reproducción asistida clínica Sagrada Familia; Centro Medico Teknon; Clínica EUGIN; Fertilab.Institut Catalá de Fertilitat; Fundació Puigvert - Hospital de la Santa Creu i Sant Pau; General Lab; Gine-3; Gravida; H. Del Mar; Hospital Clínic de Barcelona; Hospital Quiron Barcelona; Hospital Valle Hebrón; Institut Dexeus; Institut Marques; Instituto de Reproducción CEFER; IVI Barcelona; SOMDEX Dr. Santiago Dexeus; Barcelona IVF; Girexx Barcelona - Fertility Clínics SL.; Fertty

Benalmádena: IVI Malaga

Beniarbeig: IREMA

Benidorm: IMED Hospitales

Bilbao: Clínica Ginecológica Bilbao; Consultorio Ginecologico Elcano; Instituto IGIN; Quiron Bilbao; Reproducción BIlbao

Boadilla del Monte: HM Fertility Center Montepríncipe

Burjassot: UR IMED Valencia.

Cáceres: Clínica Norba

Cádiz: Clínica La Salud; Grupo Médico de Reproducción, gmer; H. U. Puerta del Mar

Carcaixent: FIVIR

Cartagena: Instituto Bernabéu Cartagena, SL.; IVI Cartagena

Castellón: H. General De Castelllon; Hospital Rey Don Jaime; IVI Castellón

Ciudad Real: Clínica Rubal; QuirónSalud Ciudad Real

Ciutadella de Menorca: IBILAB Menorca

Córdoba: Clínica Bau - Cordoba; Clínica IFEM; Clínica Mesa Dominguez Clínica MD; Clínica Povedano; H. U. Reina Sofia; Icreavida

El Ejido: Clínica JOFRE-FIV

El Palmar: Hospital Clínico Universitario Virgen de la Arrixaca

Elche: H. General De Elche; In Vitam Centro de Medicina Reproductiva

Figueres: FIV Obradors

Fuengirola: Clínica Fertia

Getafe: H. De Getafe; Instituto Para El Estudio De La Esterilidad

Gijón: FIV4-Instituto de Reproducción Humana Gijón; Clínica Ergo Biotech SL

Girona: Centro De Genética Girona; Girexx (FIV Girona SLP); Hospital Universitari De Girona Doctor Josep Trueta; Unitat de Reproduccio Humana i Diagnostìc Genètic. Clínica Girona

Guadalajara: FIV Laber

Granada: Clínica Sanabria; H.U. Virgen de las Nieves; Instituto Avantia de Fertilidad; MAR&Gen; UR HOSPITAL INMACULADA; Vithas Hospital la Salud; Aluz, Clínica de Fertilidad Avanzada; Clínica Pedrosa

Granollers: FECUNMED

Huelva: Hospital Costa de la Luz

Ibiza: Hospital Can Misses; IBILAB Pititusses

Jaén: CARHA; Ciudad de Jaén

Jerez De La Frontera: ULTRAFIV-BAHIA S.L.; Clínica Serman; Clínica Beiman

La Cañada: Unidad de Reproducción Hospital Virgen del Mar

La Laguna: Centro de Asistencia a la Reproducción Humana de Canarias; H. Universitario De Canarias

Las Palmas de Gran Canaria: CIRA Las Palmas; H.U.Materno Infantil de Las Palmas de Gran Canaria; Instituto Canario De Infertilidad, S.L.; IVI Las Palmas

Leioa: IVI Bilbao

León: Centro Asistencial Universitario de León( CAULE ); Centro Ginecológico de León

Lleida: Avantmedic Unitat de la Dona; FIv Lleida; CEFER Lleida

Logroño: Centro Ginecologico Manzanera; Clínica Alxen . Centro Ginecológico Riojano; Clínica Ginecológica Juana Hernández; Ginesalud; H. San Pedro

Lugo: EVALO Centro Ginecológico

Madrid: Centro Médico Milenium Alcobendas Sanitas; Clínica Dr. Eduardo Cubillo; Clínica Ruber Internacional; Clínica Ruber-Centro de Reproducción Madrid, S.L.; Clínica Tambre; EVA QX Lab, S.L; FivMadrid; Fundacion Jimenez Diaz UTE; GINEFIV; H. 12 De Octubre; H. Ramon y Cajal; Hospital Clínico San Carlos; Hospital General Universitario Gregorio Marañón; Hospital La Paz; Hospital Nuestra Señora de América; Hospital Universitario Moncloa S.A.U; IGMR Dres Ordás y Palomo; Instituto Europeo de Fertilidad; Instituto Ginecologico 'La Cigüeña'; Instituto Madrileño de Fertilidad; IVI Madrid; MINIFIV; Procreatec; Clínica EasyFIV; Eugin; Amnios In Vitro Projec

Málaga: Centro Gutenberg; H. Materno Infantil De Malaga - Carlos Haya; Hospital Quiron Málaga; IMAFER – Clínica Victoria; Instituto Malavé de Reproducción; Malaga F.I.V.; UR El Ángel

Marbella: CERAM (Centro De Reproducción Asistida De Marbella); FIV Marbella; HC-Fertility; Hospital Costa de Sol; Hospital Ochoa

Mataro: IMARA

Melilla: Clínica Ginecológica Dr. Marín; Clínica Imera

Móstoles: HM Fertility Center Puerta del Sur

Murcia: Centro Ginecológico de Fertilidad y Genética; Fertilidad Roca; Imar Fertilidad; Instituto de Reproducción Asistida QuironSalud Dexeus Murcia; IVI Murcia; Tahe Fertilidad; Unidad de Reproducción La Vega

Oviedo: CEFIVA - Oviedo; FIV4-Instituto de Reproducción Humana; H. UCA, Unidad reproducción, Hospital materno-infantil

Palamós: Serveis de Salut Integrats Baix Empordà

Palma De Mallorca: CEFIVBA: Centro Fecundacion In Vitro Balear; FIV de Mallorca; Hospital Universitario Son Espases; H. Fundación Son Llatzer; Instituto de Fertilidad; IVI Illes Balears

Pamplona: Complejo Hospitalario de Navarra; Estudio Médico Navarro; Quirón Pamplona

Pozuelo de Alarcón: Hospital Universitario Quirónsalud Madrid

Reus: Biogest; Conceptum; Procrear

Rincón De La Victoria: Instituto de Fertilidad Clínica Rincón

Roquetas De Mar: Roquetas FIV

Salamanca: Clínica Mencía

Salt: Hospital de Santa Caterina. Salt

San Sebastián: Clínica Zuatzu; Hospital Quirón Donostia; Hospital Universitario de Donostia; Instituto Vasco de Fertilidad Donostia; IVI San Sebastian / Donostia

Sant cugat del Valles: Áptima Centre Clinic Mutua de Terrasa

Santa Cruz de Bezana: FIVSantander

Santa Cruz De Tenerife: Centro De Endocrinologia De La Reproducción de Tenerife; Centro Madre (Centro Mahatni de Reproducción); H. Nuestra Sra. De La Candelaria; Irmo, S.L.

Santander: CER Santander; IVI Santander; URA Valdecilla. Hospital U .Marques de Valdecilla

Santiago de Compostela: Complexo Hospitalario Universitario de Santiago; Unidad de Reproducción Asistida La Rosaleda; Zygos, Centro Gallego de Reproducción

Sevilla: Caremujer- Quiron Sagrado Corazón; Embryocenter; Ginemed; H. Universitario Virgen De Valme; Hospital Victoria Eugenia (INEBIR); Hospital Virgen del Rocío; IVI Sevilla; MasVida Reproducción; Hospital Quirón Infanta Luisa

Tarragona: Embriogyn

Toledo: H. Virgen De La Salud; HM IMI Toledo

Torremolinos: URA Clínica Santa Elena

Valencia: Clínica Quiron Valencia; CREA Valencia; Equipo Juana Crespo; FIV Valencia; H. Arnau De Vilanova; H. Dr. Peset Aleixandre; H.U. La Fe; Hospital Clínico Universitario de Valencia; Hospital General Universitario de Valencia; Imer; IVI Valencia S.L; Policlínico Valencia; UHRA Nisa-Ginemed

Valladolid: Fiv Madrid Valladolid; FIV Recoletos Valladolid; Hospital Universitario Rio Ortega; Unidad Reproducción, Servicio Ginecología y Obstetricia del H.C.U. De Valladolid

Vic: Unitat Endocrinologia Ginecològica

Vigo: Centro Hospitalario Universitario de Vigo; Centro Medico Pintado; Hospital Nuestra Señora de Fátima; IVI Vigo; Clínica Nida

Vitoria: ART Vitoria; Hospital Universitario Araba sede Txagorritxu

Zaragoza: Centro Médico Ginfer SLP; Clínica Ginecológica de Zaragoza S.L.; Clínica Gobest; Clínica Montpellier (UR Montpellier); H. Clínico Zaragoza; H. Miguel Servet; IVI Zaragoza; Quirónsalud Zaragoza

**Sweden**

Falun: Livio Falun

Göteborg: Livio Fertilitetscentrum Göteborg; Reproduktive medicin Sahlgrenska University Hospital; Nordic IVF Göteborg

Linköping: RMC Linköping University Hospital.

Malmö: Livio Malmö; RMC Malmö University Hospital; Nordic IVF Malmö

Örebro: IVF unit Örebro University Hospital

Stockholm: Livio Kungsholmen; IVF unit Karolinska University Hospital, Huddinge; IVF unit Sophiahemmet; Livio Gärdet; Stockholm IVF-clinic; Nordic IVF Stockholm, Stockholm IVF

Umeå: Livio Umeå

Uppsala: Carl von Linné clinic; Reproductive center, Academiska University Hospital

**Switzerland**

Baden: Kinderwunschzentrum Baden

Basel: Universitäts - Frauenklinik Basel; Kinderwunschzentrum Regio Basel; Fertilitas

Bellinzona: Endomed

Bern: Kinderwunschzentrum - Universitätsfrauenklinik; IVF-Team Lindenhofspital;

Biel : Care - Centrum für assistierte Reproduktionsmedizin und gynäkologische Endokrinologie

Chur: Kinderwunschzentrum Fontana

Freiburg : Centre de Procréation médiclement assistée

Genève: Centre PMA Clinique Générale Beaulieu-UNILABS; Centre Clinique des Grangettes ; Hôpitaux Universitaire de Genève; Medixy

Kreuzlingen: Milagro

Lausanne: Centre de procréation médicalement assistée (CPMA);  Unité de Médecine de la Reproduction et d’Endocrinologie gynécologique - CHUV

Locarno: Centro Cantonale di Fertilità

Lugano: ProCrea

Luzern: Kinderwunschzentrum Kantonsspital Luzern; Kinderwunschzentrum Klinik St. Anna Hirslanden

Olten: Fertisuisse

St Gallen: FIORE- Fachinstitut für Reproduktionsmedizin und gynäkologische Endokrinologie; IVF Zentrum Prof. Zech

Schwyz: Kopelli Klinik

Zug: GEA IVF

Zürich: GYN-A.R.T. AG; Kinderwunschzentrum Universitätsspital Zürich; Gyné Invitro; OVA - IVF Clinic Zürich; 360 Grad Kinderwunsch Zentrum; Admira Kinderwunschzentrum

**The Netherlands**

Amsterdam: Academisch Medisch Centrum; Vrije universiteit Medisch Centrum

Elsendorp: Nij Geertgen

Groningen: Universitair Medisch Centrum Groningen

Hengelo: Fertiliteitskliniek Twente

Leiden: Leids Universitair Medisch Centrum

Leiderdorp: Medisch Centum Kinderwens

Maastricht: Universitair Medisch Centrum Maastricht

Nijmegen: Universitair Medisch Centrum Nijmegen

Rotterdam: Erasmus Medical centre

Tilburg: Elisabeth Twee Steden Ziekenhuis

Utrecht: Universitair Medisch Centrum Utrecht

Voorburg: Reiner de Graaf Groep

Wolvega: Nij Barrahûs

Zwolle: Isala

**Ukraine**

Chernivtsi: Medical centre of infertility treatment

Dnipro: Genesis - Dnepr - IVFLTD «Medical Plaza»; LTD «Aurora IVF»

Ivano-Frankivsk: Precarpacian Centre of Reproductive Medicine; Clinic of Reproductive Medicine Extramed; Medical Reproductive Health Centre Damia

Kharkiv: Academician V.I.Grishchenko Clinic for Reproductive Medicine; “ART-clinic”; LTD “Sana-med”; State institution “Ukrainian medical center of obstetrics, gynecology and reproductology of Ministry of public health of Ukraine”; Medical Center  "Pharmacy and Medicine"

Khmelnytskyi: Olena Vavrynchuk Reproductive Clinic

Kyiv: “Mother and Child” clinic; A.A. Partners; “Rodynne dzherelo” Clinic; NADIYA Clinic of Reproductive Medicine; Isida IVF clinic; “Victoria” Reproductive Genetics Clinic; Clinic of reproductive technologies USIR SNMAPE; Ukrainian State Institute of Reproduction; Institute of Reproductive Medicine; Institute of Genetic Reproduction; “Biotexcom”; Medical Cneter “Nativita”; Institute for Family Planning; Center for Reproductive Medicine “MATERI CLINIC”; LTD "Mini-EKZ Center"; LTD “Academiс medical center”; Medical Center “IVF Laboratory”, LTD “Sana-Med Kiev”

Lutsk: Center for Reproductive Medicine BOGOLYUBY

Lviv: IVF clinic “Alternatyva”; Center for Infertility Treatment «Parens-Ukraine»; IVF clinic “Intersono”, Medical Center “Clinic of prof. S. Khmil”(Lviv), “Mother and Child” clinic (Lviv)

Odesa: REMEDI Center for Reproductive Medicine; “Gameta” Reprocuctive Health clinic; LADA Reproductive Health Clinic; Reproductive medicine department, University clinic, Odessa national medical university; Medical Center “Academium”

Rivne: Medical Center “Blagodar”

Ternopil: Medical Center “Clinic of prof. S. Khmil”

Vinnitsa: Medical Center “Vinukrmed”

Zaporizhzhia: Regional Center for rehabilitation of reproductive health

**United Kingdom**

Aberdeen: Aberdeen Fertility Centre

Bath: CARE Bath

Belfast: Regional Fertility Centre, Belfast; TFP Belfast Fertility

Birmingham: Birmingham Women's Hospital; CREATE Fertility, Birmingham; BMI The Priory Hospital; CARE Birmingham

Brighton: Brighton Fertility Associates

Brighton & Hove: Agora Clinic Brighton

Bristol: Bristol Centre for Reproductive Medicine; CREATE Fertility Bristol

Cambridge: Bourn Hall Clinic; Cambridge IVF

Canterbury: BMI The Chaucer Hospital

Cardiff: London Women’s Clinic, Wales; Wales Fertility Institute – Cardiff

Carshalton: Beginnings at Epsom & St Helier NHS University Trust

Cheshire: Hewitt Fertility Centre, Knutsford

Cheshunt: Herts and Essex Fertility Centre

Chester: CARE Fertility Chester

Colchester: Bourn Hall Clinic (Colchester)

Coventry: Centre for Reproductive Medicine, Coventry

Croydon: Fertility in Community

Daresbury: Reproductive Health Group

Darlington: London Women’s Clinic, Darlington

Derriford, Plymouth: Centre for Reproduction and Gynaecology Wales and West

Dundee: Ninewells Hospital

Eastbourne: Sussex Downs Fertility Centre

Edinburgh: Edinburgh Fertility Centre

Epsom: NewLife Fertility Centre

Essex: Simply Fertility

Exeter: Fertility Exeter

Gateshead: The Gateshead Fertility Unit

Glasgow: Glasgow Nuffield Hospital; Glasgow Royal Infirmary; TFP GCRM Fertility

Hull: Hull IVF Unit

Leeds: Leeds Fertility

Leicester: Leicester Fertility Centre; X&Y Fertility

Liverpool: Hewitt Fertility Centre

Llantrisant: Centre for Reproduction & Gynaecology Wales (CRGW)

London: Andrology Solutions; Assisted Reproduction and Gynaecology Centre; Barts Health Centre for Reproductive Medicine; Boston Place; CARE London; Chelsea & Westminster Hospital; City Fertility; Concept Fertility; CREATE Fertility, London St Paul's; CREATE Fertility, London Wimbledon; Guys Hospital; Harley Street Fertility Clinic; Homerton Fertility Centre; IVI London (Wimpole Street); King’s Fertility; London Fertility Centre; London Women's Clinic; Reproductive Genetics Institute; The Centre for Reproductive and Genetic Health; The Evewell; The Fertility & Gynaecology Academy; The Fertility Centre at Whittington Health; The Lister Fertility Clinic; Wolfson Fertility Centre - Hammersmith Hospital

Maidenhead: TFP Thames Valley Fertility

Manchester: CARE Manchester; CREATE Fertility, Manchester; Manchester Fertility; St Mary's Hospital

Middlesbrough: The James Cook University Hospital

Newcastle Upon Tyne: Newcastle Fertility Centre at Life

Northampton: CARE Northampton

Norwich: Bourn Hall Clinic Norwich

Nottingham: CARE Nottingham; NUH Life Fertility Services; TFP NURTURE Fertility

Oxford: TFP Oxford Fertility

Port Talbot: Wales Fertility Institute-Neath

Salisbury: Salisbury Fertility Centre

Sheffield: CARE Sheffield; Jessop Fertility

Shrewsbury: Shropshire and Mid-Wales Fertility Centre

South Bromley: Kent Fertility Centre

Southampton: Complete Fertility Centre Southampton; TFP Wessex Fertility

Tamworth: CARE Tamworth

Tunbridge Wells: CARE Tunbridge Wells

Wickford: Bourn Hall Clinic Wickford

Woking: CARE Woking

Wolverhampton: St Jude's Women’s Hospital
